# Genome-Wide Analysis of lncRNA-mRNA Co-Expression Networks in CD133+/CD44+ Stem-like PDAC Cells

**DOI:** 10.3390/cancers15041053

**Published:** 2023-02-07

**Authors:** Giasemi C. Eptaminitaki, Apostolos Zaravinos, Dimitris Stellas, Maria Panagopoulou, Sevasti Karaliota, Ismini Baltsavia, Ioannis Iliopoulos, Ekaterini Chatzaki, Dimitrios Iliopoulos, Stavroula Baritaki

**Affiliations:** 1 Laboratory of Experimental Oncology, Division of Surgery, School of Medicine, University of Crete, 71003 Heraklion, Greece; 2 Basic and Translational Cancer Research Center (BTCRC), Genomics and Systems Biology Laboratory, Cancer Genetics, Nicosia 1516, Cyprus; 3 Department of Life Sciences, School of Sciences, European University Cyprus, Nicosia 2404, Cyprus; 4 Institute of Chemical Biology, National Hellenic Research Foundation, 11635 Athens, Greece; 5 Laboratory of Pharmacology, Medical School, Democritus University of Thrace, 68100 Alexandroupolis, Greece; 6 Institute of Agri-Food and Life Sciences, Hellenic Mediterranean University Research Centre, 71410 Heraklion, Greece; 7 Laboratory of Computational Biology, Division of Basic Sciences, School of Medicine, University of Crete, 71003 Heraklion, Greece; 8 Athos Therapeutics Inc., Los Angeles, CA 90502, USA

**Keywords:** pancreatic ductal adenocarcinoma (PDAC), long non-coding RNAs (lncRNAs), cancer stem cells (CSCs), cancer biomarkers

## Abstract

**Simple Summary:**

The prognosis of pancreatic ductal adenocarcinoma (PDAC) remains unfavorable among PDAC patients and is accompanied by high mortality rates. Cancer stem cells (CSCs) have a main role in PDAC aggressiveness. The present study sheds light on the molecular characterization of cancer stem-like subpopulations that significantly confer to PDAC aggressiveness and identifies CSC-specific lncRNA signatures with potential prognostic and therapeutic significance in PDAC.

**Abstract:**

Pancreatic ductal adenocarcinoma (PDAC), the second most prevalent gastrointestinal malignancy and the most common type of pancreatic cancer is linked with poor prognosis and, eventually, with high mortality rates. Early detection is seldom, while tumor heterogeneity and microarchitectural alterations benefit PDAC resistance to conventional therapeutics. Although emerging evidence suggest the core role of cancer stem cells (CSCs) in PDAC aggressiveness, unique stem signatures are poorly available, thus limiting the efforts of anti-CSC-targeted therapy. Herein, we report the findings of the first genome-wide analyses of mRNA/lncRNA transcriptome profiling and co-expression networks in PDAC cell line-derived CD133+/CD44+ cells, which were shown to bear a CSC-like phenotype in vitro and in vivo. Compared to CD133−/CD44− cells, the CD133+/CD44+ population demonstrated significant expression differences in both transcript pools. Using emerging bioinformatic tools, we performed lncRNA target coding gene prediction analysis, which revealed significant Gene Ontology (GO), pathway, and network enrichments in many dyregulated lncRNA nearby (*cis* or *trans*) mRNAs, with reported involvement in the regulation of CSC phenotype and functions. In this context, the construction of lncRNA/mRNA networks by ingenuity platforms identified the lncRNAs ATF2, CHEK1, DCAF8, and PAX8 to interact with “hub” SC-associated mRNAs. In addition, the expressions of the above lncRNAs retrieved by TCGA-normalized RNAseq gene expression data of PAAD were significantly correlated with clinicopathological features of PDAC, including tumor grade and stage, nodal metastasis, and overall survival. Overall, our findings shed light on the identification of CSC-specific lncRNA signatures with potential prognostic and therapeutic significance in PDAC.

## 1. Introduction

PDAC, the most common type of pancreatic cancer, is among the deadliest malignancies, with a dismal 5% five-year survival rate after diagnosis [1]. In the last decade, PDAC incidence has been significantly raised in both genders, to the point where it is projected to become the second leading cause of cancer-related deaths by 2030 [2]. Early PDAC diagnosis is extremely difficult, as it does not show clear clinical symptoms, which delays treatment and worsens the disease prognosis [3]. PDAC aggressiveness lies mainly in complex molecular mechanisms underlying the uncontrollable tumor cell proliferation and rapid dissemination that lead to distant metastases [4]. These mechanisms include key adaptive changes in tumor metabolic and molecular signaling, cell adhesion to tumor stroma, and immune tolerance [4]. In addition, PDAC cells have developed a dense functional layer that facilitates tumor resistance to conventional chemotherapy and radiotherapy [5].

Recent advances in tumor biology have revealed the presence of a small group of cells within tumors, known as cancer stem cells (CSCs), which are of particular research interest. According to the CSC concept that was proposed four decades ago, tumor growth is fueled by small numbers of dedicated stem cells. Over the years, it has become clear that most solid tumors harbor CSCs which have the capacities of plasticity, quiescence, unlimited self-renewal, and tumor initiation exclusively in vivo [6]. The existence of CSCs within the bulk of the tumor not only sustains tumor growth but also promotes metastasis and tumor resistance to conventional chemotherapies [7]. As such, CSCs are highlighted by experts as the ideal therapeutic targets in most solid tumors once we understand the molecular mechanisms that regulate their key biological properties.

The pancreatic CSCs (PCSCs) account for less than 2% of the total PDAC cell number [8]. To date, the cellular membrane markers that have been identified and used for selective identification and isolation of PCSCs include CD133, CD24, CD44, CXCR4, EPCAM, ESA, Nestin, c-Met, Aldh1, DclK1, and Lgr5 [9,10,11,12]. These markers can be detected individually or in combination on PCSC membranes and contribute to PCSC properties, including tumor resistance to chemotherapy [7,13]. However, the complete and necessary co-existence of all the above different markers has not been reported in PCSCs. Previous studies have also shown overexpression of some PCSC markers (CD24, CD44, Dclk1, CXCR4, ESA, and Nestin) in low and high pancreatic intraepithelial lesions (PanIN), indicating that PCSCs may also be involved in the gradual transition of PanIN to PDAC [9,12,14]. The main signaling pathways reported in the literature to positively regulate the characteristic properties of PCSCs are the: (1) Hedgehog, which activates the expression of Oct4, Nanog, c-Myc, and Sox2 known to be responsible for CSCs pluripotency [15,16]; (2) Notch, which also maintains the molecular features of PCSCs involved in self-renewal, including the expression of CD44, EpCAM, Oct4, Nanog, and PDX1 markers [17,18]; and last (3) PI3K/AKT which stimulates the proliferation of PCSCs, mainly of those expressing CD133 [19]. The Wnt pathway is also considered a PCSC-specific marker as it serves as a regulator of Lgr5 gene expression; however, its role in PCSC properties is less understood. Overall, the identification, molecular characterization, and therapeutic targeting of CSCs, including PCSCs, have not been as obvious as initially projected due to the lack of specific and reliable tumor-associated CSC biomarkers [7]. Therefore, advances in the field of PCSC biomarker development are emerging.

In recent years, the research on biomarker development in various diseases, including cancer, has shown a particular orientation toward the so-called “non-coding” regions of our genome. Thanks to the availability of innovative technologies, it is now evident that while less than 2% of the total human genome encodes proteins, at least 75% is actively transcribed into non-coding (nc) RNA molecules [20]. The majority of this, 75%, includes long (>200 nucleotides in length) ncRNAs, known as lncRNAs [21]. LncRNAs are expressed in a tissue-specific and controllable manner, while they are involved in the regulation of gene expression at epigenetic, transcriptional, and post-transcriptional levels through a variety of mechanisms [22]. Many of the lncRNA target genes have direct implications in cancer [21]. Functional studies have revealed the correlation of lncRNA levels with the expression patterns of discrete groups of genes that control either transformation of healthy cells into cancer or affect basic properties of transformed cancer cells, such as tumor cell proliferation, survival, metastasis, and response to host immune surveillance, or conventional chemotherapeutics [21]. In addition, lncRNA dyregulation has been clinically associated with poor prognosis of various cancer types [23]. LncRNAs are, therefore, functional RNA transcripts with the ability to regulate the basal properties of cancer cells, and consequently, their potential therapeutic targeting is of research and clinical interest [24]. LncRNA expression patterns and lncRNA/mRNA interacting networks in PDAC remain largely unexplored. LncRNA transcriptome profiling may allow the identification of lncRNAs that may affect at different levels nearby coding genes with a catalytic role in PDAC pathophysiology, thus serving as early diagnostic, prognostic biomarkers as well as therapeutic targets in the most aggressive malignancy of the pancreas [25].

Just over the last few years, the hypothesis of the possible involvement of lncRNAs in the regulation of CSC properties at different molecular bases, including interaction with other macromolecules at the epigenetic, transcriptional, and post-transcriptional levels, has started to gain ground [26]. However, the direct association of specific lncRNAs with diverse CSC populations through complex lncRNA/mRNA interacting networks has not been clearly elucidated in PDAC [23]. In this context, in the present study, we have attempted a transcriptome profiling analysis, at mRNA and lncRNA levels, in a CD133+/CD44+ PDAC subpopulation that bears functional features of CSCs. To this end, we set four specific aims: (1) to validate in our experimental setting the CD133+/CD44+ PDAC cell stemness phenotype in vitro and in vivo, (2) to detect in a genome-wide microarray assay dysregulated patterns of mRNA and lncRNA expression in our study population and establish through bioinformatic analysis possible associations with CSC-related biological processes and molecular functions, (3) to construct lncRNA-mRNA co-expression networks and predict CSC-associated target coding genes that may be targeted by dysregulated lncRNAs in CD133+/CD44+ PDAC cells, and (4) to identify possible correlations of “node” lncRNAs with clinicopathological characteristics of PDAC. Overall, our study contributes to the efforts of identifying and utilizing lncRNAs as putative CSC biomarkers of diagnostic and therapeutic significance in PDAC.

## 2. Materials and Methods

### 2.1. Cell Lines and Culture Conditions

The human PDAC cell lines MIA PaCa-2 and PANC-1, purchased from American Type Culture Collection [(ATCC), Manassas, VA], were used in the study. Both cell lines were cultured in Dulbecco’s Modified Eagle’s Medium (DMEM, Invitrogen, MA, USA) supplemented with 10% (*v*/*v*) heat-inactivated fetal bovine serum (FBS), 10 U/mL penicillin, and 100 μg/mL streptomycin (all from Gibco, Carlsbad, CA, USA). The medium for MIA PaCa-2 culture was further supplemented with horse serum to a final concentration of 2.5%. Cells were incubated at 37 °C with 5% CO_2_. All procedures involving cell lines of human origin were approved by the Institutional Biosafety Committee.

### 2.2. Cell Sorting

Isolation of CD133+/CD44+ and CD133−/CD44− cells from MIA PaCa-2 and PANC-1 PDAC cell lines was performed in a BD FACs Aria Cell Sorter (BD Pharmingen, San Jose, CA, USA) using a 100 nozzle. Briefly, for each cell line, 3 × 10^7^ cells grown as monolayers in TC-treated dishes at 80–90% confluence were harvested with Accutase (Sigma-Aldrich, Inc., Milwaukee, WI, USA), washed with Hank’s Balanced Salt Solution (HBSS, StemCell Technologies, Kent, WA, USA) and split in 3 polypropylene FACs tubes containing 100 μL cold staining buffer (HBSS + 2% BSA). Cells were then sequentially incubated in ice with 20 μL FITC-conjugated CD133 mAbs (BD Pharmingen) for 15 min and 5 μL of anti-CD44-PE mAb (BD Pharmingen) for 10 more minutes. Following two washes with cold HBSS, the cells were resuspended in 500 μL HBSS supplemented with 2% FBS and analyzed. Discrimination of dead cells was performed by addition of 5 μL 7-AAD/tube 5 min before sorting. Tubes containing untreated cells, single stained cells with the above mAbs, or FITC and PE isotype controls were used. The purity of each isolated population ranged between 85 and 95%.

### 2.3. Tumorsphere Formation and Passaging

The ability of tumorsphere formation was assessed in sorted CD133+/CD44+ and CD133−/CD44− cell subpopulations of MIA PaCa-2 and PANC-1. Briefly, single-cell suspensions were plated in ultra-low attachment 6-well plates (Corning, New York, NY, USA) at a seeding density of 15,000 viable cells/well and cultured in 2 ml/well serum-free complete MammoCult medium (StemCell Technologies) containing MammoCult Basal Medium, MammoCult Proliferation (1/10 dilution), fresh Hydrocortisone (0.48 μg/mL) and Heparin (4 μg/mL) (all from StemCell Technologies). Tumorspheres larger than 60 μm were imaged and counted after cell incubation at 37 °C, 5% CO_2_ for 7 days. For passaging, tumorspheres that had not developed a dark center were collected by gentle centrifugation (350 g) after 7–10 days of culture and dissociated both enzymatically and mechanically for 10 min using pre-warmed Trypsin-EDTA (0.05% trypsin, 0.53 mM EDTA-4NA) and 23-gauge needle, respectively. Following a single cell wash with HBSS + 2% FBS, single-cell suspensions were cultured in complete MammoCult medium, as previously described, for the generation of subsequent tumorspheres.

### 2.4. Cytotoxicity Assay

Isolated CD133+/CD44+ and CD133−/CD44− cells, as well as unsorted (total) MIA PaCa-2 and PANC-1 cells grown as monolayers in TC-treated dishes at 80–90% confluence, were single-treated in 96-well plates with various concentrations of Gemcitabine for 48 h. All cell treatments were performed in media containing 0.1% FBS, and cell viability was assessed by XTT according to the manufacturer’s instructions (Invitrogen, Waltham, MA, USA).

### 2.5. Superarray Analysis for Inflammation and EMT Markers

Total RNA was extracted from MIA PaCa-2- and PANC1-derived CD133+/CD44+ and CD133−/CD44− cell populations using the RNeasy Plus Mini Kit (Qiagen, Germantown, MD, USA). cDNA was synthesized using the quantitative reverse-transcription kit (Qiagen). The expression profiles of 168 genes, of which 84 were related to epithelial-to-mesenchymal transition (EMT) and the remaining 84 to inflammation, were determined using 96-well format RT^2^ Profiler PCR arrays (PAHS-011Z; PAHS-090Z; Qiagen). The PCR reactions were set up using RT^2^ SYBR Green qPCR Mastermix (SABiosciences/Qiagen) and run on an ABI 7500 Fast qPCR instrument (Applied Biosystems, MA, USA). Data analysis was performed using the 2−ΔΔCt method described on the manufacturer’s website (https://www.qiagen.com/gr/resources/resourcedetail?id=20762fd2-8d75-4dbe-9f90-0b1bf8a7746b&lang=en, accessed on 18 June 2021). Heatmaps of the relative gene expression were generated using heatmap v0.2.4 in R with the *ward.D2* cluster method and Euclidean distance. Venn diagrams were used to find commonly dysregulated mRNAs/lncRNAs between PANC-1 and MIA PaCa-2 cells using the program Venn2 (v0.1.0) in R.

### 2.6. In Vivo Studies

#### 2.6.1. Xenograft Mouse Models

NOD. Cg-Prkdcscid Il2rgtm1Wjl/SzJ female mice were purchased from The Jackson Laboratory, ref RRID: IMSR_JAX:005557. Matrigel, an extract of basement membrane proteins that form a 3D gel at 37 °C, was used as cell carrier medium to facilitate the cell inoculation into the mice. Briefly, unsorted (total) and sorted CD133+/CD44+ and CD133−/CD44− cell populations derived from the cell lines MIA PaCa-2 and PANC-1 were mixed with Matrigel (Corning, AZ, USA) at a concentration of 8 × 10^5^ cells/100 μL, diluted 1:3 in Matrigel and subsequently injected into the flanks of anesthetized 6–8-week-old mice. As soon as tumors reached measurable size, their size was assessed by external measurement of their length (l) and width (w) using a Vernier caliper. Accordingly, the tumor volume (mm^3^) was calculated using the following equation: (l × w2) × π/6. Statistical analyses and graph design were performed by GraphPad Prism 9.2.0 (San Diego, CA, USA). Tumor volumes were plotted as mean with standard deviation for each data point. The animal study protocol was approved by the local ethics committee (Athens Prefecture Veterinarian Service; K3237/11-05-2019) and took place in the animal facilities of the Institute of Chemical Biology of the National Hellenic Research Foundation (NHRF). All animals were handled in strict accordance with good animal practice as defined by the relevant European and Greek animal welfare bodies. The animal facility of NHRF is operating under ISO (9001:2015) for the following scope: animal model unit operation and surgical protocols, in vivo experiments, and xenografts. Registration number: I-030-02-100-01430, valid until 15-06-2025.

#### 2.6.2. Histology and Immunohistochemistry Staining

The excised tumors were fixed overnight in 10% neutral buffered formalin in PBS, followed by paraffin embedding and microtome sectioning. Five μm tumor sections were subjected to immunohistochemistry after dewaxing, rehydration, and hematoxylin and eosin (H&E) staining. Briefly, antigen retrieval was performed by heat-inactivation, peroxidase activity inhibition by 1.5% hydrogen peroxide, and blocking with mouse serum 2% in PBS. Cell proliferation was calculated by IHC, using a mouse anti-human mAb against Ki67 (Cell Signaling Technology, Inc., Danvers, MA, USA) (1:1000 dilution), followed by incubation with an HRP-conjugated anti-mouse secondary Ab (Sigma-Aldrich). Ki67-positive and Ki67-negative cells were counted using ImageJ software (US National Institutes of Health) by staining three different slides per tumor, one from the first one-third of each tumor, one from the middle section, and one slide derived from the lower one-third of each tumor.

### 2.7. Gene Microarray Assay and Data Analysis

For each cell line MIA PaCa-2 and PANC-1, two pairs of CD133+/CD44+ and CD133−/CD44− cell samples, isolated by different sortings, were used for microarray analysis (total 8 samples, 4 compared pairs, each consisting of one double positive and one double negative cell sample). Total RNA from each sample was extracted as described above, quantified using a NanoDrop 2000 spectrophotometer (Thermo Fisher Scientific, Inc.), and amplified using TargetAmp™ 1-Round aRNA Amplification Kit (Epicentre). For microarray analysis, Agilent Array platform was employed. The sample preparation and microarray hybridization were performed based on the manufacturer’s standard protocols with minor modifications. The labeled cRNAs were hybridized onto a third-generation Human LncRNA Microarray V3.0 (8*60K, Arraystar), capable of detecting 30,586 human LncRNAs and 26,109 protein-coding transcripts that cover the most highly-respected public transcriptome databases (Refseq, UCSC known genes, Gencode, etc.), as well as landmark publications. Each transcript was represented by a specific exon or splice junction probe, which could identify individual transcripts accurately. Positive probes for housekeeping genes and negative probes were also printed onto the array for hybridization quality control. The arrays were scanned, and the acquired images were analyzed by Agilent Feature Extraction software (version 11.0.1.1). Quantile normalization and subsequent raw data processing were performed using the GeneSpring GX v11.5.1 software package. Differentially expressed LncRNAs and mRNAs between compared samples (CD133+/CD44+ vs. CD133−/CD44−) of each cell line pair were identified through Fold Change (FC) filtering (FC ≥ 1.5 and adj. *p*-value < 0.04 were set as a cut-off value). Volcano plots were constructed after normalization and standardization of the obtained values and used to visualize differentially expressed genes with statistical significance (*p* ≤ 0.5), while hierarchical clustering (HCl) analysis was performed to show the distinguishable lncRNAs and mRNAs expression patterns among tested samples. Commonly dysregulated mRNA genes between the groups of differentially expressed mRNAs and predicted lncRNA-targeted mRNAs in CD44+/CD133+ cells were calculated for each cell line and presented in Venn diagrams using the software Venn2 (v0.1.0).

### 2.8. Bioinformatic Analysis

#### 2.8.1. Gene Ontology (GO) and Reactome Pathway Analyses

GO terms were used to annotate and classify gene function. The differentially expressed genes were put into the Database for Annotation, Visualization, and Integrated Discovery v6.8 (DAVID; http://david.abcc.ncifcrf.gov/, accessed on 8 January 2023), which utilizes GO to identify the (1) Biological Processes (BP), (2) Cellular Components (CC), and (3) Molecular Functions (MF) associated with the gene profile [27]. Furthermore, we used ConsensusPathDB-Human Release 34 (cpdb.molgen.mpg.de, accessed on 5 July 2021) [28] to perform Reactome analysis, which was applied to determine the potential roles of the differentially expressed mRNAs that may play in biological pathways. A *p* < 0.05 was set as a cut-off value.

#### 2.8.2. Ingenuity Pathway and Network Analysis

Ingenuity Pathway Analysis (IPA; Qiagen) was performed to identify the canonical pathways and the co-expression networks formed by the lncRNAs and mRNAs (mRNAs/mRNAs, lncRNAs/mRNAs, and lncRNAs/lncRNAs), based on experimentally observed results [29]. The causal networks of these differentially expressed molecules were created based on their connectivity score. We also investigated the biological functions linked with the genes in each network.

#### 2.8.3. In Silico Data Analysis and Clinicopathological Associations

The expression of the lncRNAs identified to interact with “hub” SC-related mRNAs was correlated with PDAC clinicopathological characteristics, including tumor grade and stage, nodal metastasis, and overall patients’ survival, using an in silico approach. RNA-seq data (read counts) for the genes ATF2, CHEK1, DCAF8, and PAX8 were extracted from the TCGA-PAAD project of the Cancer Genome Atlas using the Genomic Data Commons (GDC) Data Portal (https://portal.gdc.cancer.gov/, accessed on 8 July 2022). Read counts were then normalized to log_2_ transcripts per million (TPM) mapped reads, as previously described [30]. Gene expression-related comparisons were made between primary pancreatic adenocarcinoma (PAAD) and normal samples, as well as across the different cancer stages, grades, and lymph node metastasis. A *p*-value < 0.05 was considered as threshold for statistical significance. We also used Kaplan–Meier curves to plot overall survival in patients with high or low expression of the genes of interest, using the median expression as cut-off. The log-rank test with HR and 95% CI was used for analysis. Adjusted *p*-values < 0.05 were considered statistically significant.

### 2.9. Statistical Analysis

Statistical analysis was performed using GraphPad Prism Software (version 5; GraphPad Software, Boston MA, USA). Student’s *t*-test or Mann–Whitney U test was applied to compare the variables of two groups, while differences between multiple groups were determined using analysis of variance (ANOVA). For correlation analysis, Spearman and Pearsons tests were applied. Survival plots were generated by Kaplan–Meier analysis, and the log-rank test was used to assess the significance of the differences. Uni-variate analysis was also performed to assess the predictive value of the expression of the ATF2, CHEK1, DCAF8, and PAX8 lncRNAs using SPSS Statistics (v29.0). Results were considered statistically significant if *p* ≤ 0.05. Values are expressed as the mean ± standard error of the mean (SEM).

## 3. Results

### 3.1. CD133+/CD44+ PDAC Cells Harbor CSC Characteristics In Vitro

**Figure 1:**
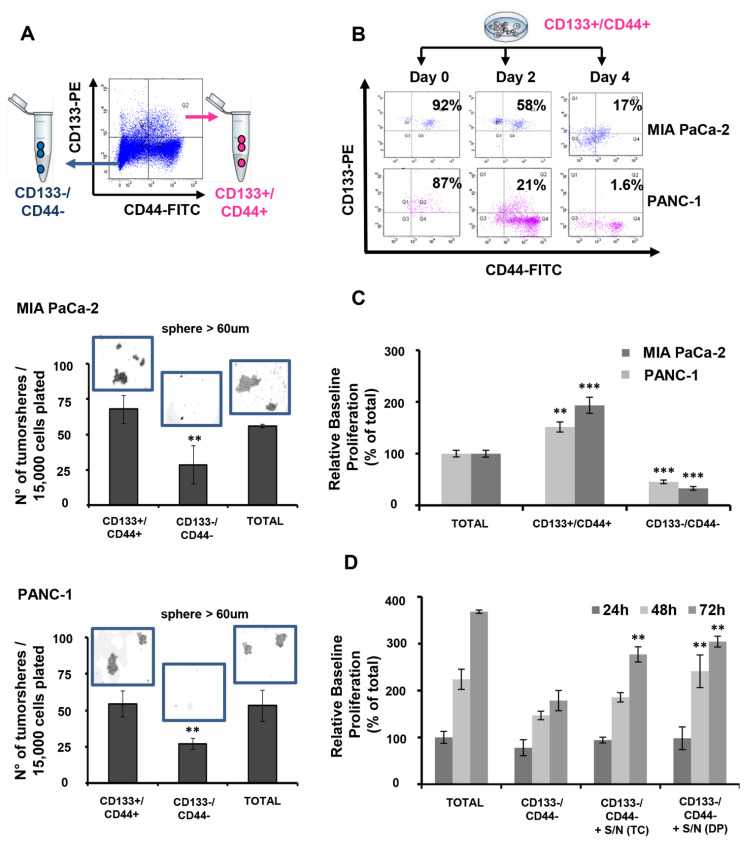
CD133+/CD44+ PDAC cells harbor CSC characteristics. (**A**) Isolated CD133+/CD44+ (+/+) PDAC cells form more tumorspheres and are of larger size compared to those formed by isolated CD133−/CD44− (−/−) PDAC cells. Both cell sub-populations were isolated by MIA PaCa-2 and PANC-1 PDAC cell lines using flow cytometry and immediately cultured in Mamocult medium for seven days. Tumorspheres larger than 60 μm were counted and photographed on day 7 from wells containing the isolated and total cells. (**B**) CD133+/CD44+ PDAC cells are able to establish the initial tumor heterogeneity. All four cell phenotypes were significantly enriched following culture of MIA PaCa-2-derived CD133+/CD44+ PDAC cells for up to 4 days. Representative flow cytometry plots were captured and shown at days 0, 2, and 4. (**C**) CD133+/CD44+ PDAC cells show significantly increased basal cell proliferation compared to CD133−/CD44− cells. Data shown are retrieved after 72 h of cell culture in complete medium. (**D**) CD133−/CD44− cells significantly increase their baseline proliferation upon co-culture with CD133+/CD44+ (+/+) and unsorted total cell (TC)-derived supernatants (S/N) for 48 and 72 h. ** *p* < 0.01, *** *p* < 0.001.

To access in vitro the CSC-specific self-renewal ability of the MIA PaCa-2- and PANC-1-derived CD133+/CD44+ and CD133−/CD44− populations, isolated cells were cultured in Mamocult medium for tumorsphere formation. At day 7 post-culture, double positive cells had formed significantly more and larger size tumorspheres compared to those formed by CD133−/CD44− PDAC cells (Figure 1A). Notably, no significant difference was observed in the number and size of tumorspheres formed by double positive and total cells, thus suggesting that although the percentage of the CD133+/CD44+ cells within the total cell population ranges only between 1 to 2.5%, it contributes drastically to the functional phenotype of each cell line, as it relates to self-renewal ability. Single positive populations for each marker were also able to form tumorspheres; however, their numbers were significantly lower than those of double positive and total cells (Supplementary Figure S1A). Accordingly, all four cell phenotypes were significantly enriched following culture of isolated CD133+/CD44+ PDAC cells for up to 4 days, therefore indicating their ability to establish the initial tumor heterogeneity in vitro (Figure 1B). Contrarily, this is not evidenced for double negative or single positive PDAC sub-populations even after 10 days of culture (Supplementary Figure S2). Furthermore, CD133+/CD44+ PDAC cells showed significantly elevated basal cell proliferation compared to those of total or CD133−/CD44− cells; (Figure 1C) however, the latter could be notably improved upon co-culture with supernatants derived by double positive or total cell cultures (Figure 1D).

**Figure 2:**
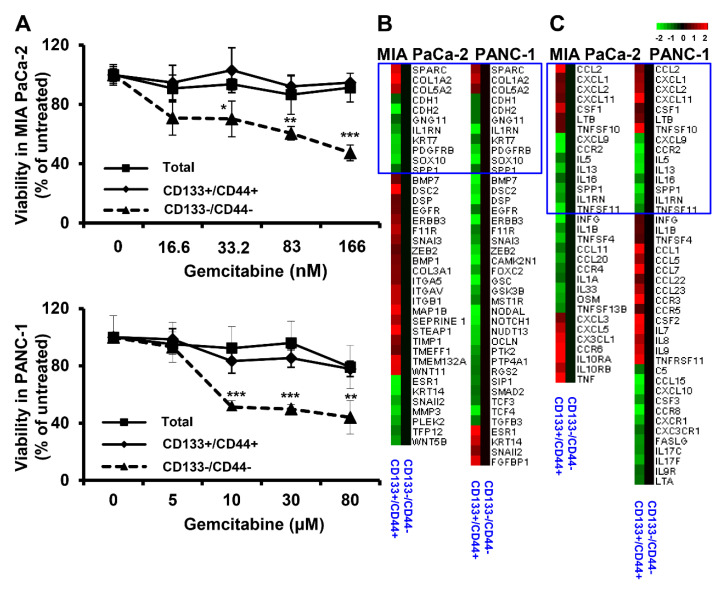
Double-positive cells show increased resistance to Gemcitabine and differential expression of genes associated with inflammation and EMT regulation. (**A**) CD133+/CD44+ PDAC cells are significantly more resistant to treatment with Gemcitabine than CD133−/CD44− cells. The indicated cells were isolated by MIA PaCa-2 and PANC-1 cell lines and cultured for 48 h in presence of various concentrations of Gemcitabine before assessment of cell viability by XTT. (**B**,**C**) Differential expression of EMT- and inflammation-related gene markers in MIA PaCa-2 and PANC-1-derived double- positive compared to double-negative cells, respectively. The expression was assessed by SuperArray analysis using RT^2^ Profiler PCR array platforms. The square areas indicate commonly dysregulated genes between the two cell lines. Each gene marker was considered to be differentially expressed when the fold change (FC) was >2 between the compared cell populations. * *p* < 0.05, ** *p* < 0.01, *** *p* < 0.001.

Evaluation of the cell response to conventional PDAC chemotherapy with gemcitabine showed significantly higher sensitivity of double negative populations to various drug concentrations than double positive and total cells, whereas no significant differences were recorded between the drug responses of CD133+/CD44+ and total cells (Figure 2A). Moreover, differential expression of EMT- and inflammation-related gene markers were observed in both cell lines, between double positive and double negative cells, as assessed by SuperArray assays (Figure 2B,C). Double-positive cells derived by both cell lines shared commonly dysregulated genes with the same expression patterns for EMT and inflammation markers. Specifically, the increased expression of SPARC, COL1A2, and COL5A2 and the decreased expression of CDH1, IL1RN, and SPP1 promote EMT. Major initiators of tumor inflammation such as CCL2, CXCL1, CXCL2, CXCL11, CSF1, LTB, and TNFSF10 were found to increase in both cell lines, while inflammation suppressors like IL13and IL1RN were decreased. The lack of further similarity in the expression of the rest of the EMT and inflammation markers tested may be attributed to distinct cell line phenotypes related to tumor aggressiveness.

Overall, the above findings indicate that although both cell lines contain a high percentage of CD133−/CD44− cells which range from 30 to 50%, their aggressive phenotype may be largely attributed to the presence of the underrepresented CD133+/CD44+ PDAC subpopulation, as it harbors CSC characteristics, including abilities of self-renewal, the establishment of tumor heterogeneity, drug resistance, and dysregulated expression of EMT and inflammation markers.

### 3.2. CD133+/CD44+ PDAC Cell Populations Are Highly Tumorigenic In Vivo

**Figure 3:**
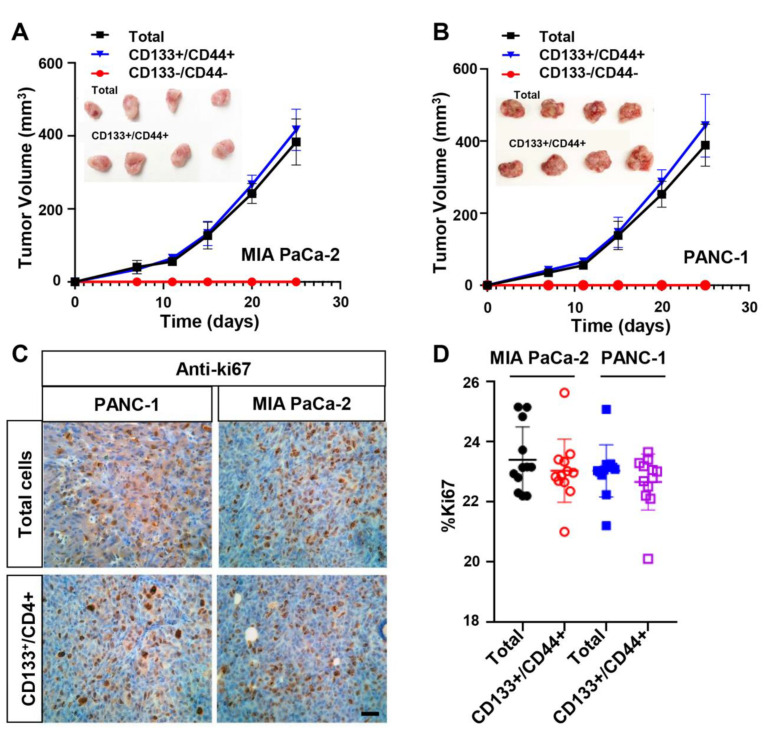
CD133+/CD44+ PDAC cells are highly oncogenic in vivo. 8×105 CD133+/CD44+, CD133−/CD44− or total (unsorted) cells derived from (**A**) MIA PaCa-2 and (**B**) PANC-1 cell lines were inoculated heterotopically into the flanks of NGS mice, and the tumor growth was monitored. CD133+/CD44+ cells show similar growth curves with total cells, whilst the sorted cells fail to grow. Tumor volumes were plotted as mean +/− SEM for each data point retrieved by two independent experiments (N = 8–10 mice/per group/experiment). (**C**) Representative immunohistochemical staining for Ki67 expression in tumors formed by MIA PaCa-2- and PANC-1-derived CD133+/CD44+ and total cells. Scale Bar = 200 μm. (**D**) The corresponding percentages of Ki67-positive cells indicate no statistically significant differences in proliferation rate between CD133+/CD44+ and total cells from both cell lines. Data were collected through three independent experiments.

To access in vivo the oncogenic ability of CD133+/CD44+ cells, total (unsorted) MIA PaCa-2 and PANC-1 cells, CD133+/CD44+ and CD133−/CD44− sorted cells were injected into NSG mice. The oncogenic potential and the size of the formed tumors were monitored for 25–27 days. Both MIA PaCa-2- and PANC-1-derived total and CD133+/CD44+ cells showed similar growth patterns, while the CD133−/CD44− cells failed to grow in vivo (Figure 3A,B). Single CD133 or CD44 positive cells also showed some, although significantly delayed, growth ability in vivo, while the size of the tumors was notably smaller than those derived by the CD133+/CD44+ cells (Supplementary Figure S3). These findings suggest that the strong oncogenic phenotype of CD133+/CD44+ cells coincides with characteristics of CSCs that may confer tumor aggressiveness.

The proliferation rates of CD133+/CD44+ and total cells were further monitored in tumor-derived sections using immunohistochemical staining for Ki67 (Figure 3C,D). The cell proliferation index was not significantly differentiated between total and double positive cells, suggesting that the underrepresented CD133+/CD44+ population may critically impact the aggressive phenotype of the whole population.

### 3.3. Differentially Expressed lncRNAs and mRNAs in CD133+/CD44+ PCSCs

**Figure 4:**
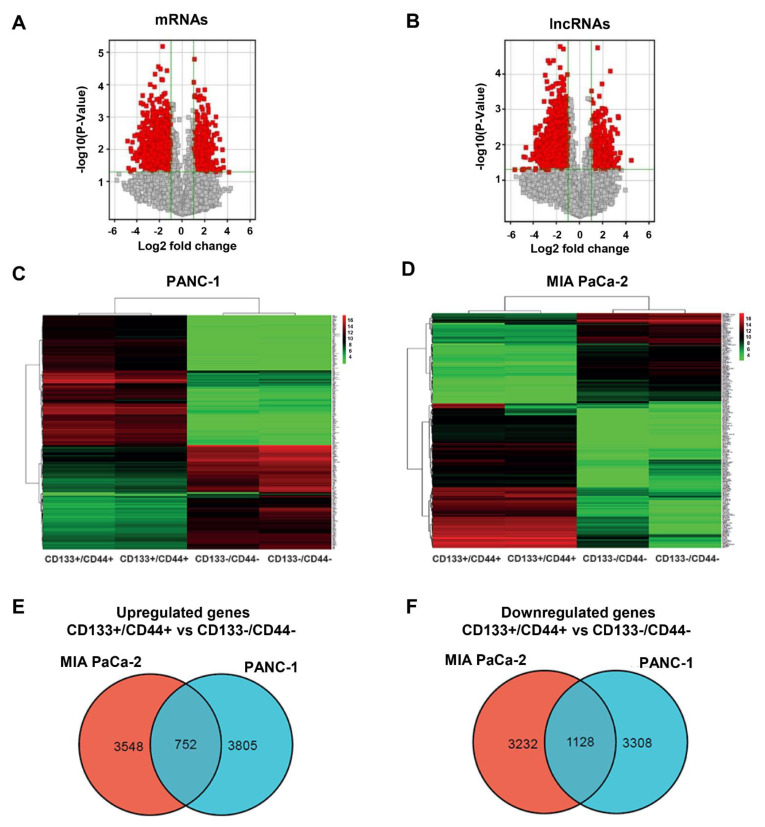
Volcano plots and HCL analysis of the dysregulated expression of mRNAs and lncRNAs and Venn diagrams of the commonly dysregulated mRNAs and lncRNAs between CD133+/CD44+ and CD133−/CD44− cell populations. (**A**,**B**) Volcano plots of the dysregulated expression of mRNAs and lncRNAs between CD133+/CD44+ and CD133−/CD44− cells. (**C**,**D**) Two-way HCl analysis (shown as heatmaps) of the differentially expressed mRNAs and lncRNAs in CD133+/CD44+ vs. CD133−/CD44− cells, derived by MIA PaCa-2 and PANC-1 PDAC cell lines, respectively. HCl analysis depicts the correlations among samples through grouping at the gene level. Each column represents one sample, and each row one gene. Red and green color grades correspond to the significantly upregulated (>1.5 fold) or downregulated (<0.5 fold) genes, respectively. (**E**,**F**) The Venn diagrams depict the common dysregulated mRNAs and lncRNAs (CD133+/CD44+ vs. CD133−/CD44−) between MIA PaCa-2 and PANC-1 PDAC cell lines.

To explore the transcriptome dyregulation in CD133+/CD44+ PCSCs, we performed a genome-wide analysis of lncRNA and mRNA expression in CD133+/CD44+ (stem cell-like) and CD133−/CD44− (non-stem cell-like) sub-populations derived by the PDAC cell lines MIA PaCa-2 and PANC-1. Volcano plotting and HCI revealed significant differences in mRNA and lncRNA expression profiles in stem cell-like compared to non-stem cell-like populations from both cell lines tested (Figure 4A–D). Specifically, in isolated CD133+/CD44+ cells from both cell lines, we identified 2628 differentially expressed lncRNAs (accounting for 8.59% of all detectable lncRNAs) and 1816 differentially expressed mRNAs (accounting for 6.96% of all detectable mRNAs). Among the dysregulated transcripts, 528 lncRNAs and 644 mRNAs were upregulated, whereas 2100 lncRNAs and 1172 mRNAs were downregulated. Interestingly, our analysis revealed various mRNAs being commonly up- or down-regulated with the predicted mRNA targets of the dysregulated lncRNAs, in CD133+/CD44+ vs. CD133−/CD44−, both in PANC-1 and MIA PaCa-2 cells, respectively (Supplementary Figure S4). We also found 752 commonly upregulated mRNAs and lncRNAs (DEGs) between MIA PaCa-2 and PANC-1 derived CD133+/C44+ cells, and 1128 commonly downregulated DEGs, respectively (Figure 4E,F). The top 20 up-/down-regulated mRNAs and lncRNAs are summarized in Supplementary Tables S1A–S2B, respectively.

### 3.4. Gene Ontology, Pathway and Network Enrichment Analyses of Dysregulated mRNAs in CD133+/CD44+ PCSCs

**Figure 5:**
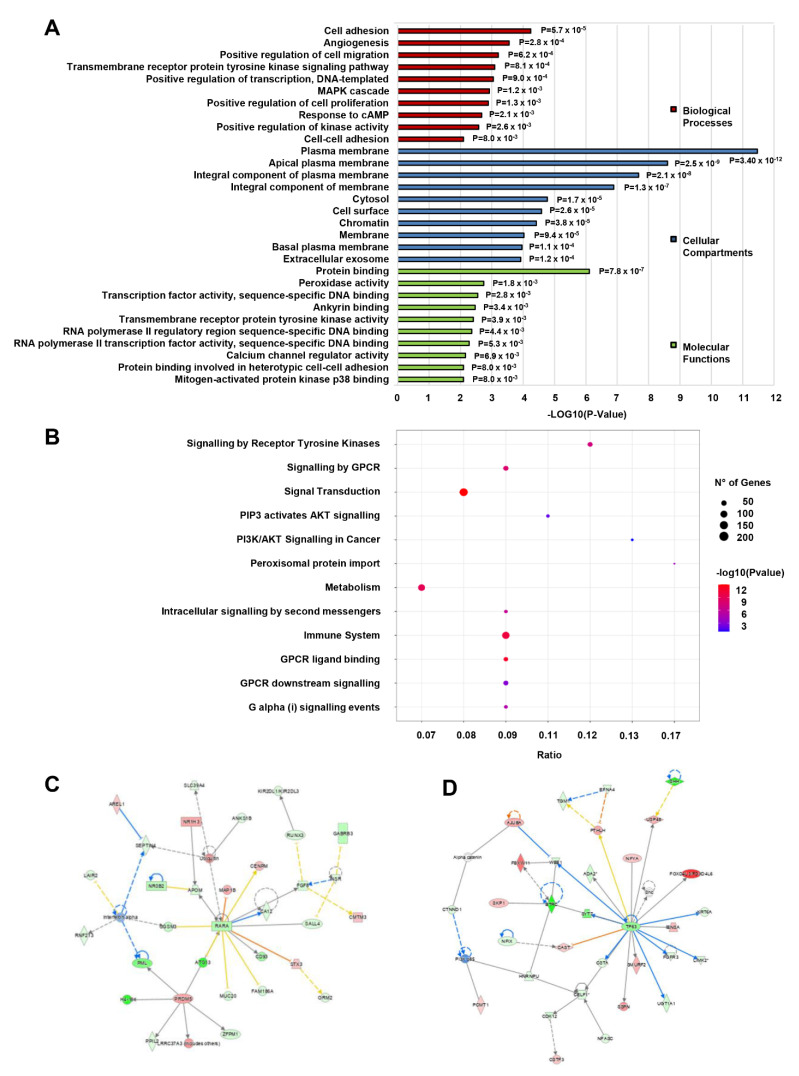
GO, pathway, and network enrichment analysis of differentially expressed mRNAs in CD133+/CD44+ PCSCs. (**A**) Gene Ontology and (**B**) Reactome pathway analysis of the differentially expressed mRNAs. The top 10 most CSC-associated enriched GO terms of each category (Biological Processes, Cellular Compartments, Molecular Functions) are summarized in (**A**). The top 12 most CSC-associated pathways are summarized in (**B**). (**C**,**D**) The top two enriched networks as assessed by IPA. The upper one associated with network functions related to “Cancer, Gastrointestinal disease, Hepatic system disease,” and the lower to “Cell cycle, Connective tissue development, and function, Connective tissue disorders”.

The differentially expressed mRNAs in CD133+/CD44+ PCSCs were processed for Gene Ontology (GO) annotation. Significant gene enrichment was assessed in molecular functions (MF), biological processes (BP), and cellular components (CC). The top BP was “cell adhesion” (GO:0007155; *p* = 5.70 × 10^−5^; 68 molecules), the top MF was “protein binding” (GO:0005515; *p* = 7.80 × 10^−7^; 1071 molecules), and the top CC was “plasma membrane” (GO:0005886; *p* = 3.40 × 10^−12^; 508 molecules) (Supplementary Tables S3A–S3C). CSC-associated functions with the most enriched genes were cell adhesion, angiogenesis, positive regulation of proliferation, migration, and cell–cell adhesion (all in BP), an integral component of the plasma membrane (in CC) and protein binding involved in heterotypic cell–cell adhesion (in MF) (Figure 5A).

The results of the Reactome pathway analysis demonstrated that differentially expressed mRNAs were mainly enriched in 37 biological pathways (Supplementary Table S4). Figure 5B summarizes the top 12 most relevant to CSC properties enriched terms that include many cancer-related metabolic pathways, e.g., ‘signal transduction’ (202 molecules), ‘GPCR ligand binding’ (43 molecules), and ‘PI3K/AKT signaling in cancer’ (14 molecules).

Likewise, Ingenuity Pathway Analysis (IPA) of the dysregulated mRNAs revealed the highest gene enrichment in molecular and cellular functions associated with “cellular movement” (*p*-value range: 2.16 × 10^−3^–3.52 × 10^−7^; 129 molecules), “cell to cell signaling and interaction” (*p*-value range: 2.34 × 10^−3^–8.36 × 10^−7^; 183 molecules), and “cell death and survival” (*p*-value: 1.98 × 10^−3^–2.43 × 10^−6^; 287 molecules), “cellular development” (*p*-value range: 2.32 × 10^−3^–3.27 × 10^−5^; 125 molecules), and “cellular growth and proliferation” (*p*-value range: 2.32 × 10^−3^–3.27 × 10^−5^; 121 molecules), while the top enriched disease was “cancer” (*p*-value range: 2.39 × 10^−3^–1.40 × 10^−51^; 1649 molecules). Accordingly, the top two associated network functions were “cancer, gastrointestinal disease, hepatic system disease” (score: 34) and “cell cycle, connective tissue development, and function, connective tissue disorders” (score: 31) (Figure 5C,D).

### 3.5. Target Coding Gene Prediction; GO Analysis, Pathway, and Network Enrichment Analyses of lncRNA-Targeted mRNAs

To explore the role of the differentially expressed lncRNAs in the regulation of CSC-associated mRNAs and metabolic pathways, the target coding genes of lncRNAs were predicted following analyses of (1) lncRNAs nearby coding gene data, (2) enhancer lncRNA profiling data, (3) enhancer lncRNA nearby coding gene data, and (4) HOX cluster profiling data. Both *cis* and *trans* lncRNA-nearby coding genes were identified in the distance < 300 kb of each lncRNA. LncRNAs with enhancer-like functions were identified using GENCODE annotation of the human genes. The consideration of a selection of lncRNAs with enhancer-like function excludes transcripts mapping to the exons and introns of annotated protein-coding genes, the natural antisense transcripts, overlapping the protein-coding genes, and all known transcripts. Furthermore, from a research and clinical perspective, enhancer lncRNAs can be considered “easier” therapeutic targets than repressor lncRNAs, since the expression of the former can be suppressed by various available means. The total number of identified lncRNAs was 2628, while the enhancer lncRNA nearby coding genes were 70. Profiling data of all probes in the four HOX loci, targeting lncRNAs and coding transcripts, were also included in the analyses.

**Figure 6:**
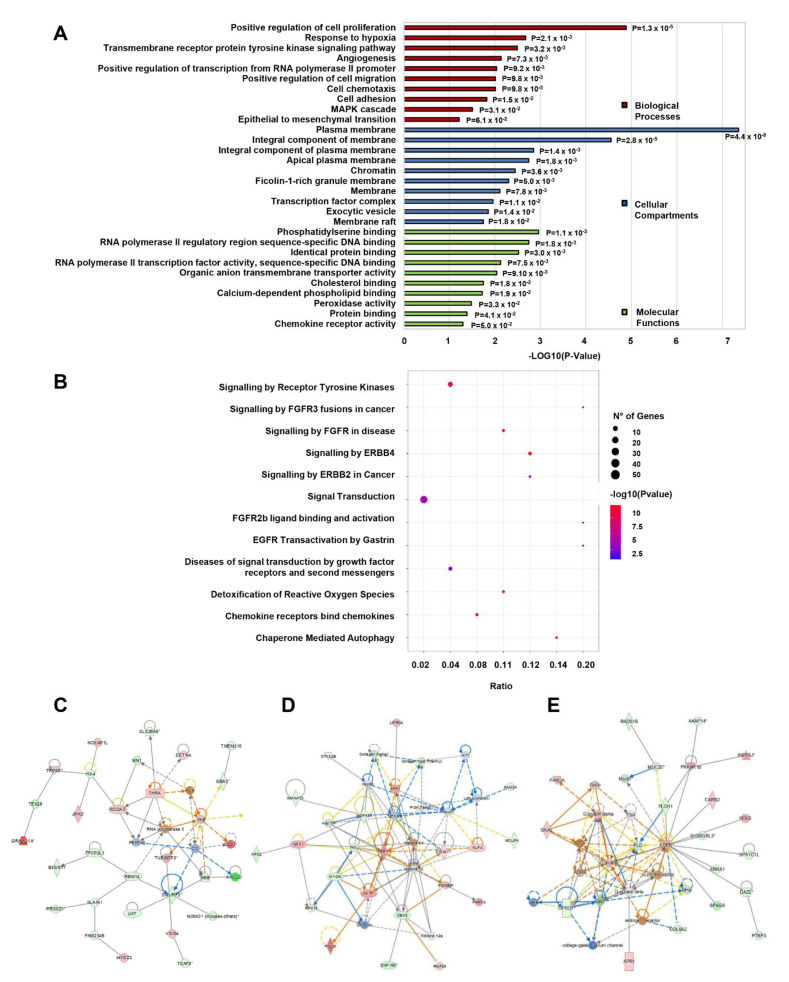
GO, pathway, and network enrichment analysis of lncRNA-targeted mRNAs in CD133+/CD44+ PCSCs. (**A**) Gene Ontology and (**B**) Reactome pathway analysis in lncRNAs’ nearby coding genes (mRNAs). The top 10 most CSC-associated enriched GO terms of each category (Biological Processes, Cellular Compartments, Molecular Functions) are shown in (**A**). The top 12 most related CSC-associated pathways are summarized in (**B**). (**C**–**E**) Highly enriched networks as assessed by IPA. The upper shows network functions associated with “cancer, gastrointestinal disease, auditory and vestibular system development, and function,” the middle with “cell death and survival, cancer, organismal injury, and abnormalities,” and the lower with “cell morphology, cell to cell signaling interaction, cellular movement”. The asterisk (*) indicates that a given gene is represented with multiple symbols.

Significant GO, pathway, and network enrichments were only assessed for the lncRNA-nearby coding gene pool. The results revealed that 2628 dysregulated lncRNAs were identified to have 595 *cis* or *trans* putative nearby target mRNAs. In the GO analysis, the top BP was “positive regulation of cell proliferation” (GO:0008284; *p* = 1.30 × 10^−5^; 27 molecules), the top MF was “phosphatidylserine binding” (GO:0001786; *p* = 1.10 × 10^−3^; 7 molecules) and the top CC was “plasma membrane” (GO:0005886; *p* = 4.40 × 10^−8^; 145 molecules) (Supplementary Tables S5A–S5C). Figure 6A summarizes the top 10 functions of each category that are associated with CSC-reported functions and properties. These include positive regulation of cell proliferation and migration, angiogenesis, response to hypoxia, cell adhesion, epithelial to mesenchymal transition (all in BP), an integral component of the plasma membrane (in CC), and chemokine receptor activity (in MF).

In the Reactome pathway enrichment analysis, the target genes were mainly enriched in 41 biological pathways (Supplementary Table S6), including many cancer-related metabolic pathways, e.g., ‘signaling by receptor tyrosine kinases’ (19 molecules), ‘signal transduction’ (52 molecules), and ‘signaling by FGFR3 fusions in cancer’ (2 molecules). The top 12 enriched pathways that contain lncRNA-nearby mRNAs that have been reported to regulate CSC-associated tumorigenesis, self-renewal, and other CSC functions are shown in Figure 6B.

IPA analysis of the dysregulated lncRNA-nearby coding genes revealed “cancer” (*p*-value range: 3.51 × 10^−4^–2.09 × 10^−29^; 406 molecules) and “gastrointestinal disease” (*p*-value range: 3.51 × 10^−4^–3.35 × 10^−17^; 366 molecules) as the most enriched disease and highest gene enrichment in molecular and cellular functions associated with “cell development” (*p*-value range: 3.24 × 10^−4^–7.37 × 10^−7^; 98 molecules), “cellular growth and proliferation” (*p*-value range: 3.24 × 10^−4^–7.37 × 10^−7^; 96 molecules), “cellular movement” (*p*-value range: 3.33 × 10^−4^–8.07 × 10^−7^; 103 molecules), “cell death and survival” (*p*-value range: 3.54 × 10^−4^–1.44 × 10^−6^; 151 molecules), and “cell-to-cell signaling and interaction” (*p*-value range: 3.29 × 10^−4^–2.74 × 10^−7^; 77 molecules). Among the 5 top enriched networks were included network functions associated with “cancer, gastrointestinal disease, auditory and vestibular system development, and function” (score: 53), “cell death and survival, cancer, organismal injury, and abnormalities” (score: 34), “cell morphology, cell to cell signaling interaction, cellular movement (score: 29) (Figure 6C–E).

### 3.6. Identification of lncRNAs Interacting with CSC-Associated mRNAs and Clinicoopathological Associations

**Figure 7:**
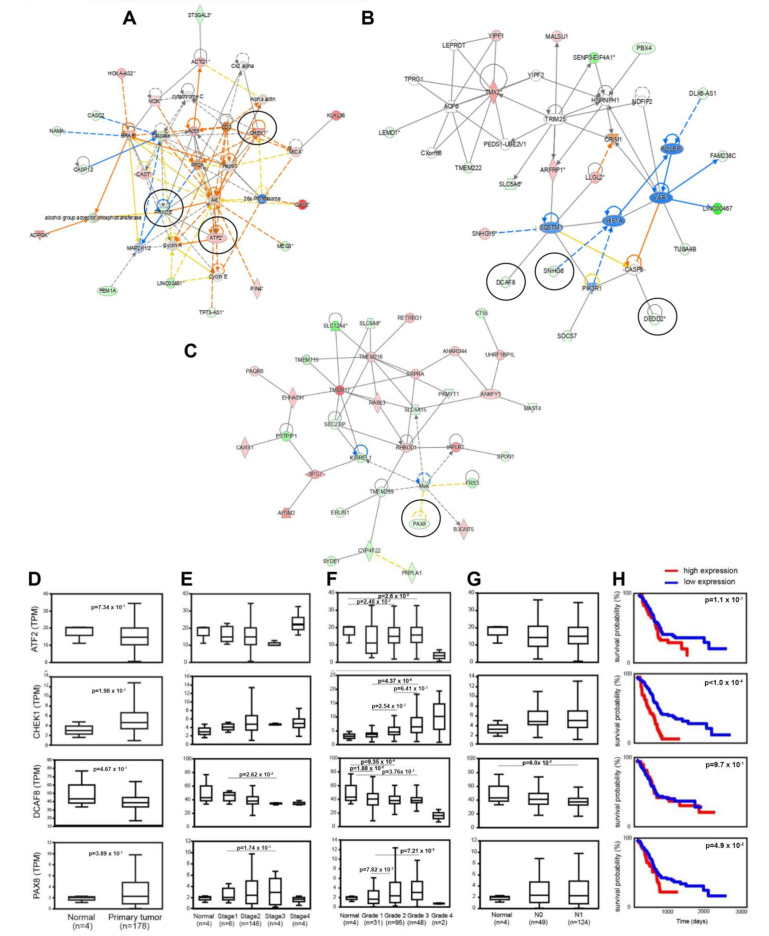
Construction of lncRNA-mRNA/lncRNA co-expression networks via IPA & clinicopathological correlations with the selected lncRNAs. (**A**–**C**) The construction of the lncRNA-mRNA/lncRNA co-expression networks revealed seven lncRNAs interacting with other lncRNAs or hub nearby mRNAs, known to be involved in CSC pathophysiology, namely ATF2, PRKCE, CHEK1, SNHG6, DEDD2, DCAF8, and PAX8 (all circled). The identified network functions are associated with “cell cycle, cell death and survival, and cellular movement” (network 1; (**A**)), “cell death and survival, cell cycle, and tissue morphology” (network 2; (**B**)) and “dermatological diseases and conditions, developmental disorder, and hereditary disorder” (network 3; (**C**)). (**D**–**H**) In silico analysis of RNA-seq data from the TCGA-PAAD project of The Cancer Genome Atlas was used for establishing gene expression differences (**D**) between PDAC and normal tissues, as well as associations with clinicopathological characteristics, including (**E**) cancer stage and (**F**) grade, (**G**) lymph node metastasis (N0/N1 nodal status) and (**H**) overall survival (depicted by Kaplan–Meier curves). The asterisk (*) indicates that a given gene is represented with multiple symbols.

Aiming to identify lncRNAs that interact with dysregulated nearby mRNAs previously reported to be involved in CSC pathophysiology or with other lncRNAs with similar functions, we constructed lncRNA-mRNA/lncRNA co-expression networks via IPA (Figure 7A–C). Seven differentially expressed lncRNAs were identified, namely ATF2, PRKCE, CHEK1, SNHG6, DEDD2, DCAF8, and PAX8, that were involved in top network functions associated with “cell cycle, cell death, and survival, cellular movement” (score: 43) (network 1; Figure 7A), “cell death and survival, cell cycle, tissue morphology” (score: 38) (network 2; Figure 7B) and “dermatological diseases and conditions, developmental disorder, hereditary disorder” (score: 43) (network 3; Figure 7C). All the identified lncRNAs showed strong interactions with nodal mRNAs, many of which have reported involvement in CSC biology. Specifically, ATF2 (Figure 7A) interacts with cyclin A, actin, and CD3, while CHEK1 (Figure 7A) with proteasome 26S, HSP90, CK2 ALPHA, cytochrome C, AKT, and PP2A. Similarly, PRKCE (Figure 7A) interacts with BRAF, AKT, HSP90, cyclin E, caspase, PP2A, as well as MAP2K1/2 and alcohol group acceptor phosphotranspherase. DCAF8 (Figure 7B) in turn links to THAP1, TFB1M, MZT2A, TUBGCP3, and SQSTM1, whereas SNHG6 (Figure 7B) interacts with HIF1A and DEDD2. Last PAX8 (Figure 7C) interconnects with the signal transducer MEK. Moreover, indirect interactions of these seven node lncRNAs with other genes were also observed.

Moreover, extracted RNA-seq data from the TCGA-PAAD project for the seven identified genes were used to perform in silico analysis for putative associations with PDAC clinicopathological parameters, including tumor grade and stage, nodal metastasis, and overall survival. Although we failed to establish significant expression differences between normal and malignant tissues (Figure 7D), a fact that might be attributed to a low number of normal samples, four out of the seven identified lncRNAs, namely ATF2, CHEK1, DCAF8, and PAX8, showed significant correlations with at least one of the clinicopathological parameters tested. The expression of DCAF8 and PAX8 in tumors illustrates patterns of significant downregulation (*p* = 0.0262) and upregulation (*p* = 0.00174) across cancer stages 1 and 3, respectively (Figure 7E). Likewise, ATF2, CHEK1, DCAF8, and PAX8 showed diverse expression across the different tumor grades (Figure 7F). Specifically, CHEK1 and PAX8 expression was increased gradually from stage 1 to stage 3 (*p* = 4.37 × 10^−6^ and *p* = 7.27 × 10^−3^, respectively). Conversely, ATF2 and DCAF8 expression decreased gradually from normal to stage 3 (*p* = 2.6 × 10^−2^ and *p* = 3.76 × 10^−3^). DCAF8 decrease was also marginally associated (*p* = 0.06) with distal lymph node metastasis (Figure 7G), while CHEK1 and PAX8 expressions were significantly correlated with worse overall patients’ survival (*p* < 0.0001 and *p* < 0.049, respectively), as evidenced by the relevant Kaplan–Meier curves (Figure 7H). The predictive value of ATF2, CHEK1, DCAF8, and PAX8 expressions was further assessed by univariate analysis. The statistically significant findings (mainly those regarding tumor grades) recapitulate the results shown in Figure 7F (Supplementary Table S7).

## 4. Discussion

The number of ncRNAs reported to be critically involved in the pathophysiology of cancer is constantly growing, thus paving the way toward the identification of novel biomarkers of diagnostic, prognostic, and therapeutic significance [23,31]. The existence of CSCs within the malignant bulk of solid tumors, including PDAC, is considered one of the major causes of therapeutic failure and disease advancement [32]. The regulatory role of a plethora of different ncRNAs in CSC-dependent oncogenesis and tumor aggressiveness is an indisputable fact [33]. Although many lncRNAs have demonstrated pivotal roles in diverse biological pathways and cellular manifestations of oncogenesis and cancer progression [34], their contribution/consideration as putative CSC-specific biomarkers remain not clearly elucidated.

Here we report the results of the first-of-its-kind genome-wide analysis of mRNA-lncRNA co-expression networks in a CD133+/CD44+ PDAC cell subpopulation isolated by the highly aggressive cancer cell lines MIA PaCa-2 and PANC-1. These cells bear a CSC-like phenotype that critically contributes to tumor aggressiveness, as revealed by our in vitro and in vivo settings. Our findings demonstrate significant expression differences in a large number of coding transcripts (mRNAs) enriched in biological functions and pathways associated with CSC features, as well as a dyregulation in the lncRNA pool expressed in our study population. Given that most lncRNAs mediate their actions mainly in coding genes located in close proximity [35], target coding gene prediction analyses revealed significant GO, pathway, and network enrichments in many dysregulated lncRNA cis or trans nearby mRNAs, with reported involvement in the regulation of CSC phenotype and functions.

Furthermore, through the construction of mRNA/lncRNA networks, we identified four lncRNAs, namely ATF2, CHEK1, DCAF8, and PAX8, with strong interactions with ‘hub’ SC-associated mRNAs and whose expressions were significantly correlated with clinicopathological features of PDAC. Briefly, ATF2 (Figure 7A) interacts with cyclin A which is known to be essential for hematopoietic and embryonal stem cells [36]. CHEK1 (Figure 7A), in turn, interconnects with multiple coding RNAs, including proteasome 26S, whose downregulation has been associated with enhanced CSC properties [37,38,39], HSP90 which cooperates with Nanog and Oct4 in preventing its ubiquitin-mediated proteasomal degradation and, thus contributing to CSC self-renewal [40,41,42,43], CK2 ALPHA that is reported to be involved in the regulation of multiple CSC-associated functions and characteristics [44,45,46,47], cytochrome C, a recently identified target for CSC therapy [48,49], AKT, a key pathway for CSC-phenotype maintenance [50,51,52], and PP2A, whose inhibition is currently used as a therapeutic strategy to target the CSCs derived by BCR-ABL+ human leukemia [53,54]. DCAF8 (Figure 7B) interacts with SQSTM1, whose expression has been associated with CSC properties in breast cancer [55]. Lastly, PAX8 (Figure 7C) interacts with MEK, which controls CSC properties mainly through the activation of the MEK/ERK signaling pathway [56,57]. Furthermore, in silico analysis revealed that among the four identified lncRNAs, DCAF8 expression is inversely correlated with tumor stage and nodal metastasis, thus suggesting its potential suppressing role in PDAC aggressiveness, likely through regulation of CSC features. In contrast, both CHEK1 and PAX8 appear to associate positively with PDAC aggressiveness and poor prognosis, based on the deleterious effects of their elevated expression in tumor grade progression and overall survival. Notably, it is worth mentioning that the lack of establishing and/or validating significant differences in the expressions of the above lncRNAs between normal and malignant tissues in our in silico analysis might be attributed to the fact that (1) the available number of normal tissue samples was small and (2) our microarray data analysis was restricted to gene expression comparisons between isolated CD133+/CD44+ CSC-like cells and CD133−/CD44− non-CSC-like cells, which represent only a small fraction of the total cell population.

Τhus far, PAX8, CHEK1, DCAF8, and ATF2 lncRNAs have not been implicated in CSC biology, while among them, only PAX8 has been reported in the literature. PAX8 polymorphisms have been suggested as risk factors of childhood acute lymphoblastic leukemia (ALL) and non-Hodgkin lymphoma [58,59], whilst the expression of specific alleles is associated with a decreased risk of developing cervical cancer [60]. Moreover, PAX8 is involved in the progression of diabetic nephropathy by targeting the miR-17-5p/STAT3 axis [61] and osteoporosis by activating the autophagy of osteoblasts via the miR-1252-5p/GNB1 axis [62]. Upon DNA damage, CHK1, the CHEK1 lncRNA-associated mRNA, enables cell cycle arrest and DNA damage repair; thus, its inhibition may confer therapeutic targeting of CSCs in many cancer types, including PDAC, colorectal cancer, prostate cancer, non-small-cell lung cancer, and acute myeloid leukemia [63,64,65,66,67,68]. Likewise, DCAF8 lncRNA-associated mRNA, DCAF8, is a ubiquitin-related gene that decreased in osteosarcoma tissues, and together with other ubiquitin-related genes (CORO6, UBE2L3, FBXL5, DNAI1) seem to play a significant role in the clinical outcome of osteosarcoma [69]. DCAF8 further promotes the degradation of myeloid leukemia factors 1 and 2 (MLF1,2), two factors that are associated with leukemia and several other malignancies, through the ubiquitin-proteasome system [70]. Last, ATF2, the associated mRNA of ATF2 lncRNA, has been implicated in the progression and therapeutic resistance of many solid cancers, including melanoma [71], breast cancer [72], hepatocellular carcinoma [73], lung cancer [74], and prostate cancer [75].

The phenotypic characterization of CSC-like subpopulations within the tumor mass by using specific and, where possible, cancer type unique markers is of critical importance for their identification, isolation, and subsequent molecular characterization at different levels. CD133 is one of the first well-characterized CSC markers, and therefore it has been widely used to identify CSCs in several solid tumors [76,77]. In contrast, CD44 standard isoform is found in most adult tissues, whereas its variant isoforms [78,79] are expressed in multiple cancers along with specific normal epithelial tissues [80,81]. CD133+/CD44+ cells phenotype has been reported to exert extensive proliferation, self-renewal, differentiation, and invasion in prostate and colorectal tumors [78,82], characteristics that corroborate the CSC phenotype. Both CD44 and CD133 are also included in the panel of markers reported to characterize the tumor-initiating cell (TIC) phenotype in PDAC [83,84,85]. In this context, Immervoll et al. have reported the existence of CD133+/CD44+ cell populations in normal pancreatic tissues, in addition to the neoplastic pancreas; however, the preferentially centroacinar localization of these double-positive cells in the normal parenchyma, suggests that this population could be of particular interest in identifying TICs within the normal pancreas [86]. Accordingly, Chen and co-workers have shown that circulating tumor cells (CTCs), labeled positive for at least one of the above TIC markers, are found in the majority of PDAC patients and are independently predictive of poor patients’ survival and disease recurrence [87]. Upregulation of CD44 variable 6 (CD44v6) in CSC subpopulations isolated by aggressive pancreatic malignancies has been positively associated with distant metastasis [88], while CD133+ pancreatic CSCs are highly resistant to standard chemotherapy and prone to give lymph node metastasis [89]. Multivariate analysis in pancreatic carcinoma further showed that tri-expression of CD133, CD44v6, and tissue factor (TF) was an independent predictor of poor survival, while this co-expression was also associated with metastasis [90]. According to other studies, CD44+/CD133+/EpCaM+ cells isolated from pancreatic cancer cell lines display a peculiar pattern of cancer stem cell-like characteristics [91], while in PDAC patients, the co-expression of CD133/CD44 in CSCs and CD204 in tumor-associated macrophages (TAMs) have been suggested as independent prediction markers for disease-free survival [92,93]. In this line, the functional properties of our isolated CD133+/CD44+ subpopulation from PDAC cell lines support their CSC-phenotype, and they are in accordance with the aforementioned reports by other groups.

To our knowledge, this is the first report of a whole-genome mRNA-lncRNA co-expression network analysis in PDAC CSC populations; however, similar studies have been carried out in other cancer types. When and his colleagues were able to identify circRNAs, miRNAs, and mRNAs with key roles in retaining the stem cell phenotype of a CD133+/CD144+ CSC population isolated by human laryngeal squamous cell carcinoma cell lines after the construction of mRNA-miRNA-circRNA regulatory networks and functional enrichment analysis of the key genes [94]. Likewise, mRNA-miRNA regulatory networks constructed by microarray mRNA and miRNA expression data from CD133+/CD144+ CSCs isolated by human colorectal cancer cell lines revealed 31 downregulated miRNAs with suggested implication in the regulation of stem cell differentiation [95]. Accordingly, mRNA-lncRNA co-expression network and functional analyses in a CD44+CD24− breast cancer CSC population uncovered the lncRNA lncCUEDC1 as a negative regulator of stemness through inhibition of NANOG-associated biological functions [96], while the essential role of lncRNA lncTCF7 in retaining CSC self-renewal and tumor propagation properties was defined in relevant studies in CD13−CD133+ CSCs from hepatocellular carcinoma [97]. In this line, transcriptome data derived from the TCGA base and/or patients’ samples have been previously used to construct similar mRNA-lncRNA co-expression networks in an effort to identify stemness-related mRNAs and lncRNAs with diagnostic and prognostic value in various cancers, including breast cancer [98]. Specifically, the analysis of mRNAs-lncRNAs networks in glioblastoma tissue-derived CD133+/Nestin CSCs and their differentiated derivatives revealed three pairs of lncRNAs and their targeted mRNAs that may critically affect CSC differentiation [99]. Likewise, mRNA, lncRNA, and circRNA transcriptomics and relevant bioinformatic analysis in CD44+/BCMab1+ CSCs isolated from human bladder cancer specimens identified CircRNA_103809 as an important regulator of CSC features, while the functional analysis of the lncRNAs-mRNAs and circRNAs-mRNAs co-expression networks uncovered key transcripts of potential prognostic significance in bladder cancer [100].

## 5. Conclusions

Overall, our findings clearly align with the current efforts in identifying molecular signatures of CSCs with prognostic and therapeutic importance in cancer, with an emphasis on the utilization of emerging bioinformatic tools. Given the aggressiveness and the limited therapeutic responses of PDAC tumors, our study and associated findings are highly innovative in terms of the studied (1) cancer cell type, (2) CSC population that is identified by us and others to significantly confer to PDAC-stemness and overall tumor aggressiveness, and (3) lncRNA transcriptome profiling and its contribution in the hitherto unclear PDAC CSC biology. However, we acknowledge that future studies validating the expression of the identified lncRNAs ex vivo, as well as their role at a functional level, might be necessary to strengthen their suggested significance as potential CSC biomarkers in PDAC.

## Data Availability

Whole genome mRNA and lncRNA raw expression data for this study were generated by ArrayStar Inc. (Rockville, MD). Derived data supporting the findings of this study are available from the corresponding author upon request.

## Supplementary Materials

The following supporting information can be downloaded at: https://www.mdpi.com/article/10.3390/cancers15041053/s1, Figure S1: Single positive cells have lower tumorsphere formation ability than double positive cells; Figure S2: Single positive cells could not induce initial tumor heterogeneity, compared to double positive cells; Figure S3: Single CD133 or CD44 positive MIA PaCa-2 cells show delayed growth ability in vivo, while they give rise to significantly smaller size tumors compared to those derived by the CD133+/CD44+ cells; Figure S4: Venn diagrams depicting the commonly up- or down-regulated mRNAs between the groups of differentially expressed mRNAs and mRNAs predicted to be targeted by nearby lncRNAs in MIA PaCa-2 and PANC-1 cells; Table S1A: Top 20 upregulated mRNAs; Table S1B: Top 20 downregulated mRNAs; Table S2A: Top 20 upregulated lncRNAs; Table S2B: Top 20 downregulated lncRNAs; Table S3A: GO analysis of mRNAs (Biological Processes); Table S3B: GO analysis of mRNAs (Cellular Compartments); Table S3C: GO analysis of mRNAs (Molecular Functions); Table S4: Reactome pathway analysis of mRNAs; Table S5A: GO analysis of lncRNA-targeted mRNAs (Biological Processes); Table S5B: GO analysis of lncRNA-targeted mRNAs (Cellular Compartments); Table S5C: GO analysis of lncRNA-targeted mRNAs (Molecular Functions); Table S6: Reactome pathway analysis of lncRNA-targeted mRNAs; Table S7: Uni-variate analysis.
