# Supplementary material for: Genome-Wide Analysis of lncRNA-mRNA Co-Expression Networks in CD133+/CD44+ Stem-like PDAC Cells
Source: Cancers (Basel). 2023 Feb 7;15(4):1053. doi: 10.3390/cancers15041053 (PMC9954787; doi:10.3390/cancers15041053)
Supplement: Supplementary file 1 [file cancers-15-01053-s001.zip › Supplementary Figures.pdf]

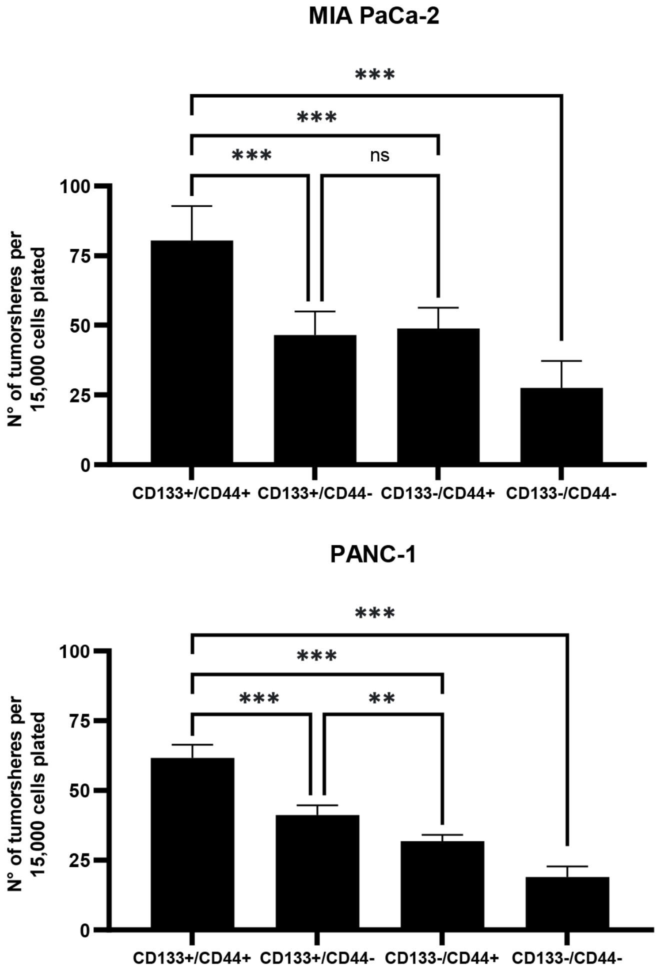

Supplementary Figure S1. Single positive cells have lower tumorsphere formation ability, than double positive cells. Isolated CD133+/CD44+ PDAC cells form more tumorspheres compared to single positive PDAC cells, CD133+/CD44- and CD133-/CD44+. Cell sub-populations were isolated by MIA PaCa-2 and PANC-1 PDAC cell lines using flow cytometry and immediately cultured in Mamocult medium for seven days. Tumorspheres larger than 60μm were counted and photographed at day 7 from wells containing the isolated and total cells. ns: not statistically significant, **p<0.01, ***p <0.001

Isolated Population	Purity (Mean ±SEM)		
	Day 0	Day 10	P-Value
CD133-/CD44-	88.3±3	non viable	
CD133+/CD44-	87± 6.5	83.4±10.6	ns
CD133-/CD44+	88.3±3	82±9.3	ns

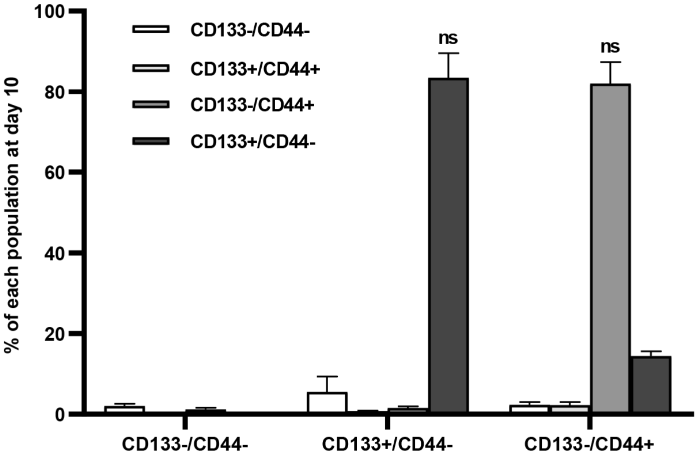

Supplementary Figure S2. Single positive cells could not induce initial tumor heterogeneity, compared to double positive cells. Isolated single positive (CD133+/CD44- or CD133-/CD44+) MIA Paca-2 cells are not as potent inducers of initial tumor heterogeneity as CD133+/CD44+ cells *in vitro* even after of 10 days in cell culture, while the double negative cells (CD133-/CD44-) fail to grow and enrich other cell phenotypes at 10 days post culture. ns: not statistically significant

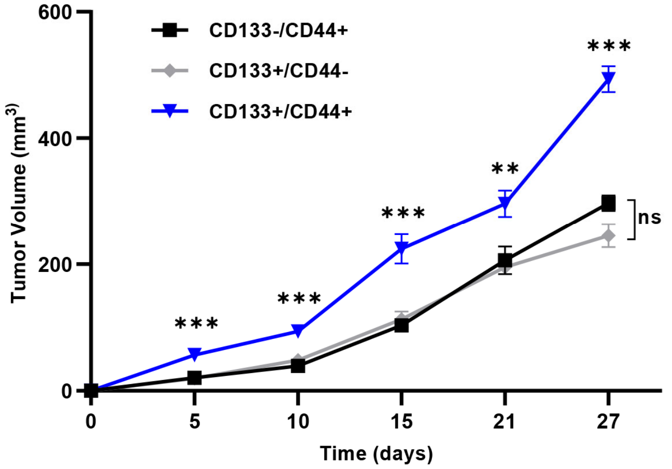

Supplementary Figure S3. Single CD133 or CD44 positive MIA PaCa-2 cells show delayed growth ability *in vivo*, while they give rise to significantly smaller size tumors compared to those derived by the CD133+/CD44+ cells. Tumor volumes were plotted as mean +/- SEM for each data point retrieved by two independent experiments (N=5-8 mice/per group/experiment). ns: not statistically significant, **p<0.01, ***p <0.001

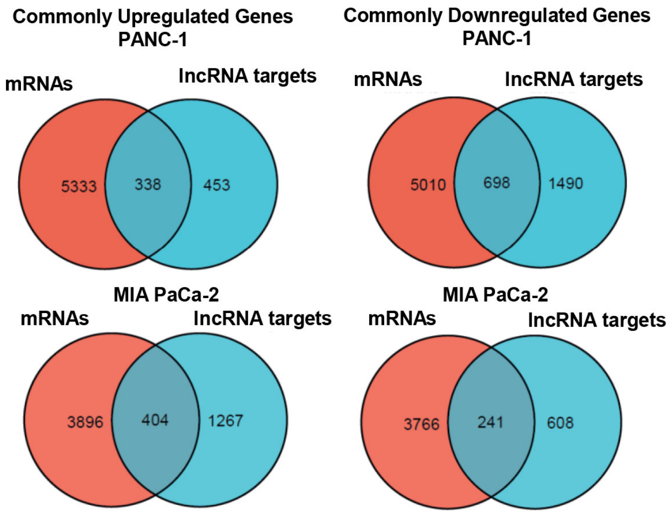

Supplementary Figure S4. Commonly up- or down-regulated mRNAs and predicted lncRNA target mRNAs, in CD133+/CD44+ vs CD133-/CD44- PANC-1 and MIA PaCa-2 cells, respectively.
