# Supplementary material for: Genome-Wide Analysis of lncRNA-mRNA Co-Expression Networks in CD133+/CD44+ Stem-like PDAC Cells
Source: Cancers (Basel). 2023 Feb 7;15(4):1053. doi: 10.3390/cancers15041053 (PMC9954787; doi:10.3390/cancers15041053)
Supplement: Supplementary file 1 [file cancers-15-01053-s001.zip › Supplementary Tables.pdf]

Supplementary Table S1A: Top 20 upregulated mRNAs

GeneSymbol	Ensembl ID	FCAbsolute	P-Value
FUNDC1	NM_173794	17.46068	0.049689
C16orf62	NM_020314	12.29326	0.035624
CCL20	NM_001130046	11.99816	0.022389
PROCR	NM_006404	11.68729	0.009918
FOXD4L3	NM_199135	10.25536	0.048714
MARCH6	NM_005885	9.907125	0.004754
GOLGA8B	NM_001023567	9.840634	0.039858
UBE2D1	NM_003338	9.176474	0.042515
TMEM218	NM_001080546	9.01993	0.046334
GOLGA8A	NM_181077	8.423025	0.003204
SC5DL	NM_001024956	8.124076	0.025484
ARL17A	NM_001113738	7.898461	0.021757
RAPGEF6	ENST00000509018	7.894114	0.048447
CAMKMT	NM_024766	7.861296	0.009222
MFSD11	NM_024311	7.742632	0.000943
TSPAN13	NM_014399	7.569858	0.025855
TMEM17	NM_198276	7.492932	0.00178
KLHL4	NM_019117	7.380729	0.041724
JAGN1	NM_032492	6.986298	0.003435
AKR1A1	NM_006066	6.886783	0.003579

*Fold Change Absolute (FCAbsolute)

Supplementary Table S1B: Top 20 downregulated mRNAs

GeneSymbol	Ensembl ID	FCAbsolute	P-Value
SHH	NM_000193	29.46876	0.005374
ZNF142	NM_001105537	27.6307	0.038263
PRDM11	ENST00000530656	24.63469	0.04641
PODXL	NM_005397	23.98378	0.009191
APCDD1	ENST00000355285	23.19588	0.01123
IL18BP	NM_001145057	21.95678	0.005911
RASL11A	ENST00000241463	21.305	0.01904
BTRC	NM_003939	20.02196	0.042187
SCD5	NM_001037582	19.30981	0.018889
FDXR	NM_004110	18.81407	0.003703
HIST4H4	NM_175054	18.24441	0.015457
ATG13	NM_014741	17.94235	0.015523
SMPD4	NM_017951	17.55455	0.012531
CBX7	NM_175709	16.99937	0.016029
LGALS12	NM_001142538	16.0268	0.021629
LILRA4	NM_012276	15.91344	0.003391
FAM175A	NM_139076	14.35496	0.004843
VASN	ENST00000304735	14.08331	0.010397
ADAM15	NM_207195	13.9844	0.002033
GRAMD1A	ENST00000317991	13.82538	0.003373

*Fold Change Absolute (FCAbsolute)

Supplementary Table S2A: Top 20 upregulated lncRNAs

GeneSymbol	Ensembl ID	FCAbsolute	P-Value	Relationship	Associated gene name
GAS5	uc001gjk.3	21.742239	0.02724765	bidirectional	ZBTB37
XLOC_004829	TCONS_00010340	11.356336	0.01648863	intergenic	
RP11-525K10.3	ENST00000568776	10.25986	0.04445846	intronic antisense	DYNLRB2
CTD-2574D22.4	ENST00000567795	10.118092	0.00167657	intron sense-overlapping	KCTD13
SNORA33	uc003qdy.1	10.0395975	0.02321029	exon sense-overlapping	RPS12
RPLP0P2	ENST00000492786	9.864012	0.00156709	intergenic	
HMlincRNA152	HMlincRNA152-	9.672991	0.00466115	intergenic	
AK130076	uc001kfc.1	9.335442	0.02656868	intron sense-overlapping	PTEN
PPP2R3B-AS1	ENST00000391707	8.193643	0.01710982	intergenic	
LINC00476	NR_023390	7.957354	0.02956955	natural antisense	ERCC6L2
BC036500	uc002xlo.3	7.8240447	0.0358034	intergenic	
SNHG1	ENST00000537024	7.5993156	0.04007331	bidirectional	SLC3A2
RP11-815I9.4	ENST00000578214	6.9221845	0.01194826	intron sense-overlapping	ZNF286B
LOC100270804	NR_026885	6.9204206	0.00747266	intergenic	
SRSF2	NR_036608	6.8999085	0.00320101	natural antisense	MFSD11
TPTE2P6	ENST00000450973	6.8918767	0.01699196	intergenic	
LOC100505815	NR_045370	6.7068377	0.02296708	intron sense-overlapping	TPD52L2
RP4-717I23.3	ENST00000440778	6.55756	0.007072	intergenic	
BC030591	uc002hkb.3	6.435237	0.005903	exon sense-overlapping	TAF15
AJ006995.3	ENST00000433344	6.43238	0.044398	intergenic	

*Fold Change Absolute (FCAbsolute)

Supplementary Table S2B: Top 20 downregulated lncRNAs

GeneSymbol	Ensembl ID	FCAbsolute	P-Value	Relationship	Associated gene name
uc.314	uc.314+	49.462475	0.048918426	intergenic	
XLOC_003052	TCONS_00007119	32.776127	0.049046054	intergenic	
ZNF300P1	uc003lta.2	29.21119	0.047347534	intergenic	
AC005324.6	ENST00000433873	29.112713	0.021507693	natural antisense	TBC1D26
XLOC_007429	TCONS_00016034	26.897802	0.04903759	intergenic	
LINC00467	ENST00000534914	25.451523	0.011703227	intergenic	
XLOC_003775	TCONS_00008931	20.762947	0.047426607	intergenic	
ANKRD30BP2	ENST00000435744	19.567062	0.025539268		
AC025165.8	ENST00000356672	17.14094	0.017815594	natural antisense	ARHGEF25
HERC2P2	ENST00000559970	16.628256	0.023619968	intergenic	
LEF1-AS1	NR_029373	16.307178	0.018945009	intergenic	
XLOC_011749	TCONS_00024431	15.143449	0.022428768	intergenic	
RP11-629G13.1	ENST00000532002	14.866012	0.004429545	natural antisense	NCAM1
RP11-40A13.1	ENST00000584688	14.681524	0.048686177	natural antisense	ASIC2
XLOC_006306	TCONS_00013650	14.441169	0.003203473	intergenic	
RP1-140A9.1	ENST00000412228	14.34874	0.022616362	bidirectional	GNB1
RP11-28B23.1	ENST00000573133	14.275999	0.01135841		
XLOC_013095	TCONS_00027580	14.225093	0.04506025	intergenic	
LINC00299	ENST00000442956	14.164954	0.00887889	intergenic	
AF131217.1	ENST00000430247	13.58193	0.014228	intergenic	

*Fold Change Absolute (FCAbsolute)

Supplementary Table S3A: GO analysis of mRNAs (Biological Processes)

Biological process	Genes Count	% Enrichment	P-Value
Cell adhesion	68	4.3	5.7E-5
Viral entry into host cell	20	1.3	1.7E-4
Angiogenesis	36	2.3	2.8E-4
Central nervous system development	25	1.6	2.9E-4
Negative regulation of cell proliferation	56	3.5	4.0E-4
Positive regulation of cell migration	36	2.3	6.2E-4
Transmembrane receptor protein tyrosine kinase signaling pathway	22	1.4	8.1E-4
Positive regulation of transcription, DNA-templated	78	4.9	9.0E-4
Ureteric bud development	10	0.6	1.0E-3
MAPK cascade	21	1.3	1.2E-3
Positive regulation of cell proliferation	62	3.9	1.3E-3
Cellular response to mechanical stimulus	16	1.0	1.8E-3
Response to camp	11	0.7	2.1E-3
Pattern specification process	9	0.6	2.4E-3
Positive regulation of kinase activity	14	0.9	2.6E-3
Positive regulation of alpha-beta T cell proliferation	5	0.3	2.8E-3
Positive regulation of T cell mediated cytotoxicity	8	0.5	2.9E-3
Hydrogen peroxide catabolic process	8	0.5	3.7E-3
Response to estradiol	18	1.1	3.7E-3
Neurotransmitter transport	10	0.6	3.8E-3
Peptidyl-tyrosine phosphorylation	21	1.3	4.6E-3
Response to cocaine	9	0.6	6.0E-3
Phosphatidylinositol biosynthetic process	10	0.6	6.1E-3
Negative regulation of interleukin-6 production	13	0.8	6.5E-3
Regulation of transcription from RNA polymerase II promoter	156	9.9	6.6E-3
ERBB4-ERBB4 signaling pathway	4	0.3	6.9E-3
Cell-cell adhesion	25	1.6	8.0E-3
Embryonic forelimb morphogenesis	8	0.5	8.0E-3
Negative regulation of T cell proliferation	10	0.6	8.1E-3
Response to oxidative stress	18	1.1	8.2E-3
Fatty acid beta-oxidation using acyl-coa oxidase	5	0.3	9.3E-3
Chemotaxis	18	1.1	9.6E-3
O-glycan processing	9	0.6	9.6E-3
Dopaminergic neuron differentiation	7	0.4	1.1E-2
Cellular sodium ion homeostasis	7	0.4	1.1E-2
Positive regulation of natural killer cell mediated cytotoxicity	7	0.4	1.1E-2
Multicellular organism development	27	1.7	1.1E-2
Cellular oxidant detoxification	13	0.8	1.1E-2
Response to drug	34	2.1	1.1E-2
Positive regulation of gamma-delta T cell differentiation	4	0.3	1.1E-2
Response to L-ascorbic acid	4	0.3	1.1E-2

Positive regulation of transcription from RNA polymerase II promoter	112	7.1	1.2E-2
Cellular response to tumor necrosis factor	20	1.3	1.2E-2
Negative regulation of tumor necrosis factor production	13	0.8	1.2E-2
Prostaglandin transport	5	0.3	1.3E-2
Ectoderm development	5	0.3	1.3E-2
T cell activation	10	0.6	1.4E-2
Adult locomotory behavior	10	0.6	1.4E-2
3'-UTR-mediated mRNA stabilization	6	0.4	1.4E-2
Positive regulation of calcium-mediated signaling	6	0.4	1.4E-2
Chemokine-mediated signaling pathway	12	0.8	1.4E-2
Positive regulation of gene expression	54	3.4	1.4E-2
Positive regulation of interleukin-2 production	8	0.5	1.5E-2
ERBB4 signaling pathway	3	0.2	1.6E-2
Cellular response to phosphate starvation	3	0.2	1.6E-2
Positive regulation of monocyte aggregation	3	0.2	1.6E-2
Protein autophosphorylation	23	1.5	1.6E-2
Carbohydrate metabolic process	22	1.4	1.7E-2
Response to isolation stress	4	0.3	1.7E-2
Negative regulation of T cell mediated cytotoxicity	4	0.3	1.7E-2
Positive regulation of smooth muscle cell apoptotic process	4	0.3	1.7E-2
Chaperone-mediated autophagy	4	0.3	1.7E-2
Response to hypoxia	23	1.5	1.7E-2
Heterotypic cell-cell adhesion	7	0.4	1.8E-2
Negative regulation of NF-kappab transcription factor activity	14	0.9	1.8E-2
Cholesterol metabolic process	13	0.8	1.9E-2
Wound healing	15	0.9	2.0E-2
Positive regulation of interleukin-1 beta production	11	0.7	2.1E-2
Endocytosis	25	1.6	2.1E-2
Response to amine	5	0.3	2.1E-2
Positive regulation of keratinocyte proliferation	5	0.3	2.1E-2
Sodium ion export from cell	5	0.3	2.1E-2
Intermediate filament organization	11	0.7	2.3E-2
Positive regulation of signal transduction by p53 class mediator	4	0.3	2.5E-2
Regulation of striated muscle contraction	4	0.3	2.5E-2
Hair follicle morphogenesis	7	0.4	2.5E-2
Adult behavior	7	0.4	2.5E-2
Dorsal/ventral pattern formation	8	0.5	2.7E-2
Positive regulation of Wnt signaling pathway	8	0.5	2.7E-2
Positive regulation of brown fat cell differentiation	5	0.3	2.7E-2
Negative regulation of cell-cell adhesion	5	0.3	2.7E-2
Positive regulation of transcription elongation from RNA polymerase II promoter	9	0.6	2.7E-2
Positive regulation of nitric oxide biosynthetic process	9	0.6	2.7E-2
Phosphatidylinositol dephosphorylation	7	0.4	2.9E-2

Proteolysis	43	2.7	2.9E-2
Positive regulation of tumor necrosis factor production	15	0.9	2.9E-2
Positive regulation of cell adhesion	10	0.6	2.9E-2
Type B pancreatic cell maturation	3	0.2	3.0E-2
CD8-positive, alpha-beta T cell activation	3	0.2	3.0E-2
Oncogene-induced cell senescence	3	0.2	3.0E-2
Peripheral nervous system neuron axonogenesis	3	0.2	3.0E-2
Amino acid transport	8	0.5	3.0E-2
Regulation of cell differentiation	8	0.5	3.0E-2
Positive regulation of interferon-gamma production	12	0.8	3.2E-2
Positive regulation of synaptic transmission, glutamatergic	7	0.4	3.3E-2
T cell migration	4	0.3	3.3E-2
Branching involved in salivary gland morphogenesis	4	0.3	3.3E-2
Hydrogen peroxide biosynthetic process	4	0.3	3.3E-2
Organic cation transport	4	0.3	3.3E-2
T cell proliferation	8	0.5	3.4E-2
Hair follicle development	8	0.5	3.4E-2
Positive regulation of ERK1 and ERK2 cascade	26	1.6	3.5E-2
Post-embryonic development	11	0.7	3.7E-2
Cell chemotaxis	11	0.7	3.7E-2
Positive regulation of peptidyl-serine phosphorylation	13	0.8	3.7E-2
Protein stabilization	25	1.6	3.8E-2
Single fertilization	11	0.7	4.0E-2
Clathrin-dependent endocytosis	6	0.4	4.0E-2
Uterus development	5	0.3	4.0E-2
Positive regulation of regulatory T cell differentiation	5	0.3	4.0E-2
Protein metabolic process	5	0.3	4.0E-2
Regulation of gene expression	28	1.8	4.0E-2
Cytokine-mediated signaling pathway	19	1.2	4.2E-2
ATP metabolic process	7	0.4	4.2E-2
Positive regulation of reactive oxygen species metabolic process	7	0.4	4.2E-2
Regulation of protein stability	13	0.8	4.3E-2
Chloride ion homeostasis	4	0.3	4.3E-2
Negative regulation of macrophage activation	4	0.3	4.3E-2
Cellular aldehyde metabolic process	4	0.3	4.3E-2
Positive thymic T cell selection	4	0.3	4.3E-2
Negative thymic T cell selection	4	0.3	4.3E-2
Mitochondrial genome maintenance	4	0.3	4.3E-2
Negative regulation of cholesterol storage	4	0.3	4.3E-2
Positive regulation of phosphatidylinositol 3-kinase signaling	12	0.8	4.3E-2
Activation of MAPK activity	11	0.7	4.3E-2
Positive regulation of wound healing	6	0.4	4.6E-2
Response to lipopolysaccharide	19	1.2	4.6E-2
Positive regulation of mononuclear cell proliferation	3	0.2	4.8E-2
Positive regulation of endothelial cell chemotaxis by VEGF-activated vascular endothelial growth factor	3	0.2	4.8E-2

receptor signaling pathway			
Positive regulation of T cell anergy	3	0.2	4.8E-2
Positive regulation of CD8-positive, alpha-beta T cell differentiation	3	0.2	4.8E-2
Negative regulation of smooth muscle cell proliferation	7	0.4	4.8E-2
Cellular response to extracellular stimulus	5	0.3	4.8E-2
Positive regulation of DNA biosynthetic process	5	0.3	4.8E-2
Positive regulation of peptidyl-tyrosine phosphorylation	13	0.8	4.9E-2
Epidermis development	12	0.8	5.0E-2
Positive regulation of interleukin-8 production	10	0.6	5.0E-2

Supplementary Table S3B: GO analysis of mRNAs (Cellular Compartments)

Cellular Compartment	Genes Count	% Enrichment	P-Value
Plasma membrane	508	32.1	3.4E-12
Apical plasma membrane	63	4.0	2.5E-9
Integral component of plasma membrane	162	10.2	2.1E-8
Integral component of membrane	486	30.7	1.3E-7
Cytosol	478	30.2	1.7E-5
Cell surface	77	4.9	2.6E-5
Chromatin	114	7.2	3.8E-5
Membrane	336	21.2	9.4E-5
Basal plasma membrane	14	0.9	1.1E-4
Extracellular exosome	209	13.2	1.2E-4
Basolateral plasma membrane	35	2.2	2.5E-4
Anchored component of membrane	18	1.1	3.1E-3
Neuron projection	44	2.8	3.1E-3
Peroxisome	18	1.1	3.7E-3
Focal adhesion	47	3.0	5.7E-3
Endoplasmic reticulum lumen	36	2.3	6.2E-3
Transcription factor complex	29	1.8	7.6E-3
Sodium:potassium-exchanging atpase complex	5	0.3	9.0E-3
Endoplasmic reticulum	106	6.7	1.1E-2
Receptor complex	27	1.7	1.1E-2
MKS complex	5	0.3	1.2E-2
Macromolecular complex	69	4.4	1.2E-2
Cytoplasm	450	28.4	1.4E-2
Exocytic vesicle	7	0.4	1.5E-2
Axon	39	2.5	1.6E-2
Melanosome	15	0.9	2.0E-2
Keratin filament	15	0.9	2.2E-2
Intrinsic component of plasma membrane	8	0.5	2.2E-2
Intrinsic component of endoplasmic reticulum membrane	4	0.3	2.4E-2
Presynapse	21	1.3	2.5E-2
Perinuclear region of cytoplasm	72	4.6	2.6E-2
Endoplasmic reticulum membrane	99	6.3	2.9E-2
Perineuronal net	3	0.2	2.9E-2
Vesicle	21	1.3	3.2E-2
Apical dendrite	5	0.3	3.2E-2
Membrane raft	28	1.8	3.4E-2
Ficolin-1-rich granule membrane	10	0.6	3.4E-2
Z disc	17	1.1	3.8E-2
Autolysosome	4	0.3	4.2E-2
Extrinsic component of membrane	11	0.7	4.4E-2
Lysosomal membrane	38	2.4	4.6E-2

Dendrite cytoplasm	7	0.4	4.6E-2
Intracellular membrane-bounded organelle	87	5.5	5.0E-2

Supplementary Table S3C: GO analysis of mRNAs (Molecular Functions)

Molecular Function	Genes Count	% Enrichment	P-Value
Protein binding	1071	67.7	7.8E-7
Cholesterol binding	13	0.8	6.6E-4
Virus receptor activity	16	1.0	8.1E-4
Peroxidase activity	10	0.6	1.8E-3
Transcription factor activity, sequence-specific DNA binding	63	4.0	2.8E-3
Ankyrin binding	7	0.4	3.4E-3
Transmembrane receptor protein tyrosine kinase activity	19	1.2	3.9E-3
RNA polymerase II regulatory region sequence-specific DNA binding	44	2.8	4.4E-3
Chaperone binding	18	1.1	5.1E-3
RNA polymerase II transcription factor activity, sequence-specific DNA binding	126	8.0	5.3E-3
Calcium channel regulator activity	9	0.6	6.9E-3
Protein binding involved in heterotypic cell-cell adhesion	4	0.3	8.0E-3
Mitogen-activated protein kinase p38 binding	4	0.3	8.0E-3
Neutral amino acid transmembrane transporter activity	6	0.4	8.1E-3
Serine-type endopeptidase activity	26	1.6	8.7E-3
Macromolecular complex binding	47	3.0	1.0E-2
Prostaglandin transmembrane transporter activity	5	0.3	1.1E-2
Chemokine receptor activity	6	0.4	1.3E-2
Phosphatidylinositol phospholipase C activity	7	0.4	1.3E-2
Collagen binding	12	0.8	1.6E-2
Transcription regulatory region sequence-specific DNA binding	29	1.8	2.0E-2
Organic cation transmembrane transporter activity	4	0.3	2.0E-2
Toxin transporter activity	4	0.3	2.0E-2
Transcriptional activator activity, RNA polymerase II transcription regulatory region sequence-specific binding	51	3.2	2.2E-2
Protein binding involved in cell-cell adhesion	7	0.4	2.3E-2
Clathrin binding	9	0.6	2.5E-2
Glutathione peroxidase activity	6	0.4	2.5E-2
Protein tyrosine kinase activator activity	5	0.3	2.5E-2
Integrin binding	21	1.3	2.7E-2
Organic anion transmembrane transporter activity	6	0.4	3.0E-2
Protein tyrosine kinase activity	16	1.0	3.0E-2
Peptidase activator activity involved in apoptotic process	3	0.2	3.3E-2
Identical protein binding	156	9.9	3.4E-2
Manganese ion binding	11	0.7	3.8E-2
Neurotransmitter transporter activity	5	0.3	4.7E-2
Adenyl-nucleotide exchange factor activity	4	0.3	4.9E-2

Ubiquitinyl hydrolase activity	4	0.3	4.9E-2

Supplementary Table S4: Reactome pathway analysis of mRNAs

Pathway name	Genes Count	% Enrichment	P-Value
Signal Transduction	202	8.3	1.92E-10
Olfactory Signaling Pathway	31	7.3	9.31E-10
Sensory Perception	44	7.5	1.68E-09
SLC-mediated transmembrane transport	34	14.0	1.28E-08
Transport of small molecules	77	12.0	1.18E-06
Developmental Biology	76	11.2	4.22E-06
Neuronal System	39	10.0	1.05E-05
GPCR ligand binding	43	9.0	1.87E-05
Immune System	162	8.9	1.96E-05
Protein localization	16	9.7	3.05E-05
Keratinization	15	11.6	6.10E-05
Transport of inorganic cations/anions and amino acids/oligopeptides	15	14.7	6.10E-05
Metabolism	137	7.0	7.92E-05
Transport of bile salts and organic acids, metal ions and amine compounds	14	16.7	1.22E-04
Class A/1 (Rhodopsin-like receptors)	29	8.4	1.24E-04
Transmission across Chemical Synapses	26	10.4	2.82E-04
Disease	91	8.8	2.86E-04
Signaling by GPCR	61	8.6	3.11E-04
Signaling by Receptor Tyrosine Kinases	56	12.1	6.03E-04
Intracellular signaling by second messengers	24	9.3	7.42E-04
G alpha (i) signalling events	27	8.5	8.28E-04
Peroxisomal protein import	11	17.2	9.77E-04
GPCR downstream signalling	55	8.7	1.10E-03
PIP3 activates AKT signaling	23	10.6	1.28E-03
PI3K/AKT Signaling in Cancer	14	13.2	2.32E-03
Neurotransmitter receptors and postsynaptic signal transmission	16	8.6	3.36E-03
Infectious disease	57	7.6	4.13E-03
Constitutive Signaling by Aberrant PI3K in Cancer	13	16.5	4.64E-03
Plasma lipoprotein assembly, remodeling, and clearance	10	16.7	5.86E-03
Peptide ligand-binding receptors	20	9.7	7.30E-03
PI5P, PP2A and IER3 Regulate PI3K/AKT Signaling	16	16.0	7.63E-03
Negative regulation of the PI3K/AKT network	16	15.0	7.63E-03
HDR through Homologous Recombination (HRR) or Single Strand Annealing (SSA)	8	6.0	7.81E-03
Homology Directed Repair	8	5.7	7.81E-03
Metabolism of lipids	46	7.1	8.14E-03
Nervous system development	45	11.9	8.51E-03
Potential therapeutics for SARS	10	12.5	9.77E-03

Supplementary Table S5A: GO analysis of lncRNA-targeted mRNAs (Biological Processes)

Biological process	Genes Count	% Enrichment	P-Value
Positive regulation of cell proliferation	27	7.0	1.3E-5
Response to drug	16	4.1	3.6E-4
Cellular response to phosphate starvation	3	0.8	1.0E-3
Chemotaxis	9	2.3	2.0E-3
Single fertilization	7	1.8	2.1E-3
Response to hypoxia	11	2.8	2.1E-3
Transmembrane receptor protein tyrosine kinase signaling pathway	9	2.3	3.2E-3
Response to estradiol	8	2.1	4.6E-3
Response to xenobiotic stimulus	12	3.1	5.3E-3
Angiogenesis	12	3.1	7.3E-3
Positive regulation of gene expression	19	4.9	8.7E-3
Response to isolation stress	3	0.8	8.8E-3
Positive regulation of transcription from RNA polymerase II promoter	35	9.0	9.2E-3
Positive regulation of cell migration	12	3.1	9.8E-3
Cell chemotaxis	6	1.6	9.8E-3
Regulation of striated muscle contraction	3	0.8	1.1E-2
Chloride transmembrane transport	7	1.8	1.1E-2
Response to cadmium ion	4	1.0	1.4E-2
Positive regulation of protein binding	6	1.6	1.4E-2
Cell adhesion	19	4.9	1.5E-2
Positive regulation of transcription, DNA-templated	23	5.9	1.5E-2
Regulation of transcription from RNA polymerase II promoter	45	11.6	1.6E-2
Cellular defense response	5	1.3	1.6E-2
Adult behavior	4	1.0	1.9E-2
Protein autophosphorylation	9	2.3	1.9E-2
Prostate gland development	3	0.8	2.0E-2
Ossification	6	1.6	2.0E-2
Positive regulation of T cell activation	4	1.0	2.2E-2
Positive regulation of synaptic transmission, glutamatergic	4	1.0	2.2E-2
Prostaglandin transport	3	0.8	2.3E-2
Positive regulation of peptidyl-serine phosphorylation	6	1.6	2.6E-2
MAPK cascade	7	1.8	3.1E-2
Response to cocaine	4	1.0	3.2E-2
Negative regulation of transcription from RNA polymerase II promoter	27	7.0	3.6E-2
Cellular response to interleukin-12	2	0.5	3.6E-2
Positive regulation of Wnt signaling pathway	4	1.0	3.7E-2
Positive regulation of cell-cell adhesion	3	0.8	3.8E-2
Regulation of cell differentiation	4	1.0	3.9E-2
Chemokine-mediated signaling pathway	5	1.3	4.0E-2
Viral entry into host cell	6	1.6	4.0E-2
Positive regulation of regulatory T cell differentiation	3	0.8	4.2E-2

Positive regulation of epithelial cell proliferation	5	1.3	4.3E-2
G-protein coupled receptor signaling pathway	26	6.7	4.5E-2
Activation of MAPK activity	5	1.3	4.5E-2
Response to organonitrogen compound	3	0.8	4.7E-2
Multicellular organism development	9	2.3	4.7E-2
Peptidyl-tyrosine phosphorylation	7	1.8	4.9E-2

Supplementary Table S5B: GO analysis of lncRNA-targeted mRNAs (Cellular Compartments)

**Cellular Compartment**	**Genes Count**	**% Enrichment**	**P-Value**
Plasma membrane	145	37.5	4.4E-8
Integral component of membrane	135	34.9	2.8E-5
Integral component of plasma membrane	43	11.1	1.4E-3
Apical plasma membrane	17	4.4	1.8E-3
Chromatin	33	8.5	3.6E-3
Ficolin-1-rich granule membrane	6	1.6	5.0E-3
Membrane	89	23.0	7.8E-3
Transcription factor complex	11	2.8	1.1E-2
Exocytic vesicle	4	1.0	1.4E-2
Membrane raft	11	2.8	1.8E-2
Pericentric heterochromatin	4	1.0	1.8E-2
Neuron projection	14	3.6	2.2E-2
Early endosome membrane	9	2.3	2.4E-2
Extracellular exosome	54	14.0	2.4E-2
Focal adhesion	15	3.9	2.5E-2
Cytoplasm	120	31.0	2.7E-2
Cytosol	118	30.5	3.2E-2
Mitochondrial outer membrane	9	2.3	3.3E-2
Intrinsic component of plasma membrane	4	1.0	3.4E-2
Extrinsic component of cytoplasmic side of plasma membrane	5	1.3	3.6E-2
Presynapse	8	2.1	3.7E-2
Ruffle membrane	6	1.6	3.7E-2
Glutamatergic synapse	15	3.9	4.0E-2
Tertiary granule membrane	5	1.3	4.4E-2
Extrinsic component of membrane	5	1.3	4.6E-2
Cell surface	19	4.9	4.8E-2
Centrosome	17	4.4	4.9E-2

Supplementary Table S5C: GO analysis of lncRNA-targeted mRNAs (Molecular Functions)

Molecular function	Genes count	% Enrichment	P-value
Phosphatidylserine binding	7	1.8	1.1E-3
RNA polymerase ii regulatory region sequence-specific DNA binding	17	4.4	1.8E-3
Identical protein binding	50	12.9	3.0E-3
RNA polymerase ii transcription factor activity, sequence-specific DNA binding	38	9.8	7.5E-3
Organic anion transmembrane transporter activity	4	1.0	9.1E-3
Virus receptor activity	6	1.6	1.6E-2
Cholesterol binding	5	1.3	1.8E-2
Calcium-dependent phospholipid binding	5	1.3	1.9E-2
Prostaglandin transmembrane transporter activity	3	0.8	2.1E-2
Peroxidase activity	4	1.0	3.3E-2
Complement component c4b binding	2	0.5	3.8E-2
Pyrimidine nucleoside transmembrane transporter activity	2	0.5	3.8E-2
Protein binding	257	66.4	4.1E-2
Metal ion binding	64	16.5	4.2E-2
Carbohydrate binding	9	2.3	4.2E-2
Chemokine receptor activity	3	0.8	5.0E-2

Supplementary Table S6: Reactome pathway analysis of lncRNA-targeted mRNAs

Pathway name	Gene Counts	% Enrichment	P-Value
Signaling by Receptor Tyrosine Kinases	19	4.1	7.93E-05
Signaling by ERBB4	7	12.1	2.54E-05
EGFR Transactivation by Gastrin	2	20.0	9.44E-03
Signaling by FGFR3 fusions in cancer	2	20.0	9.44E-03
Abacavir transport and metabolism	2	20.0	9.44E-03
FGFR2b ligand binding and activation	2	20.0	9.44E-03
Diseases of signal transduction by growth factor receptors and second messengers	10	3.6	8.85E-03
Transport of bile salts and organic acids, metal ions and amine compounds	5	6.0	8.83E-03
Transport of small molecules	18	2.8	8.48E-03
Transcriptional regulation of granulopoiesis	3	11.1	7.61E-03
Class A/1 (Rhodopsin-like receptors)	12	3.5	6.40E-03
Signaling by ERBB2 KD Mutants	3	12.0	6.12E-03
Signaling by ERBB2 in Cancer	3	12.0	6.12E-03
Organic cation transport	2	25.0	5.99E-03
Signal Transduction	52	2.1	4.50E-03
Peptide ligand-binding receptors	9	4.3	4.25E-03
Chaperone Mediated Autophagy	3	13.6	4.24E-03
SHC1 events in ERBB2 signaling	3	13.6	4.24E-03
Signaling by ERBB2 TMD/JMD mutants	3	13.6	4.24E-03
SLC-mediated transmembrane transport	10	4.1	3.89E-03
Synthesis of 15-eicosatetraenoic acid derivatives	2	33.3	3.27E-03
Chemokine receptors bind chemokines	5	8.1	2.41E-03
Norepinephrine Neurotransmitter Release Cycle	3	16.7	2.35E-03
Abacavir transmembrane transport	2	40.0	2.21E-03
Detoxification of Reactive Oxygen Species	4	11.4	1.84E-03
Neurexins and neuroligins	5	8.8	1.66E-03
GRB2 events in ERBB2 signaling	3	18.8	1.65E-03
Dopamine clearance from the synaptic cleft	2	50.0	1.34E-03
Neutrophil degranulation	17	3.5	1.14E-03
Neurotransmitter release cycle	5	10.0	9.15E-04
Neuronal System	15	3.8	8.91E-04
Signal regulatory protein family interactions	3	23.1	8.69E-04
Organic cation/anion/zwitterion transport	3	23.1	8.69E-04
Interferon alpha/beta signaling	6	8.3	7.54E-04
Cellular response to chemical stress	7	7.1	7.47E-04
Signaling by FGFR in disease	5	10.6	6.87E-04
Enzymatic degradation of dopamine by COMT	2	66.7	6.75E-04
Enzymatic degradation of Dopamine by monoamine oxidase	2	100.0	2.27E-04
Na+/Cl- dependent neurotransmitter transporters	4	21.1	1.64E-04

Supplementary Table S7: Uni-variate analysis. Uni-variate analysis assessing the predictive value of the expression of the lncRNAs ATF2, CHEK1, DCAF8 and PAX8. The statistically significant findings (mainly those regarding tumor grade) are highlighted in yellow.

Between-Subjects Factors

		N
pathologic_T		1
	T1	7
	T2	24
	T3	146
	T4	3
	TX	1

Descriptive Statistics

Dependent Variable: ATF2

pathologic_T	Mean	Std. Deviation	N
	10.61000	.	1
T1	11.38429	.814839	7
T2	11.01217	.585803	24
T3	11.08108	.685170	146
T4	11.10000	.043589	3
TX	11.34000	.	1
Total	11.08280	.668959	182

Tests of Between-Subjects Effects

Dependent Variable: ATF2

Source	Type III Sum of Squares	df	Mean Square	F	Sig.
Corrected Model	1.047[a]	5	.209	.461	.805
Intercept	1753.041	1	1753.041	3859.019	<.001
pathologic_T	1.047	5	.209	.461	.805
Error	79.952	176	.454		
Total	22435.787	182			
Corrected Total	80.999	181			

a. R Squared = .013 (Adjusted R Squared = -.015)

Between-Subjects Factors

		N
pathologic_N		1
	N0	50
	N1	122
	N1b	4
	NX	5

Descriptive Statistics

Dependent Variable: ATF2

pathologic_N	Mean	Std. Deviation	N
	10.54000	.	1
N0	11.04838	.724073	50
N1	11.12089	.656327	122
N1b	10.64050	.680912	4
NX	10.96000	.318826	5
Total	11.08280	.668959	182

Tests of Between-Subjects Effects

Dependent Variable: ATF2

Source	Type III Sum of Squares	df	Mean Square	F	Sig.
Corrected Model	1.389[a]	4	.347	.772	.545
Intercept	1995.371	1	1995.371	4436.393	<.001
pathologic_N	1.389	4	.347	.772	.545
Error	79.610	177	.450		
Total	22435.787	182			
Corrected Total	80.999	181			

a. R Squared = .017 (Adjusted R Squared = -.005)

Between-Subjects Factors

		N
pathologic_M	M0	80
	M1	5
	MX	97

Descriptive Statistics

Dependent Variable: ATF2

pathologic_M	Mean	Std. Deviation	N
M0	11.10639	.737132	80
M1	11.44000	.590635	5
MX	11.04494	.611147	97
Total	11.08280	.668959	182

Tests of Between-Subjects Effects

Dependent Variable: ATF2

Source	Type III Sum of Squares	df	Mean Square	F	Sig.
Corrected Model	.822[a]	2	.411	.917	.402
Intercept	5064.319	1	5064.319	11306.371	<.001
pathologic_M	.822	2	.411	.917	.402
Error	80.177	179	.448		
Total	22435.787	182			
Corrected Total	80.999	181			

a. R Squared = .010 (Adjusted R Squared = -.001)

Between-Subjects Factors

		N
pathologic_T		1
	T1	7
	T2	24
	T3	146
	T4	3
	TX	1

Descriptive Statistics

Dependent Variable: CHECK1

pathologic_T	Mean	Std. Deviation	N
	8.95400	.	1
T1	8.83114	.784777	7

T2	8.57854	.823591	24
T3	8.82288	.821561	146
T4	8.65300	.854538	3
TX	7.90700	.	1
Total	8.78387	.816694	182

Tests of Between-Subjects Effects

Dependent Variable: CHECK1

Source	Type III Sum of Squares	df	Mean Square	F	Sig.
Corrected Model	2.099[a]	5	.420	.623	.683
Intercept	1060.601	1	1060.601	1573.565	<.001
pathologic_T	2.099	5	.420	.623	.683
Error	118.626	176	.674		
Total	14163.179	182			
Corrected Total	120.725	181			

a. R Squared = .017 (Adjusted R Squared = -.011)

Between-Subjects Factors

		N
pathologic_N		1
	N0	50
	N1	122
	N1b	4
	NX	5

Descriptive Statistics

Dependent Variable: CHECK1

pathologic_N	Mean	Std. Deviation	N
	8.15000	.	1
N0	8.87182	.773841	50
N1	8.79150	.828497	122
N1b	8.83425	.353809	4
NX	7.80460	.766635	5
Total	8.78387	.816694	182

Tests of Between-Subjects Effects

Dependent Variable: CHECK1

Source	Type III Sum of Squares	df	Mean Square	F	Sig.
Corrected Model	5.601[a]	4	1.400	2.153	.076
Intercept	1219.179	1	1219.179	1874.448	<.001
pathologic_N	5.601	4	1.400	2.153	.076
Error	115.124	177	.650		
Total	14163.179	182			
Corrected Total	120.725	181			

a. R Squared = .046 (Adjusted R Squared = .025)

Between-Subjects Factors

		N
pathologic_M	M0	80
	M1	5
	MX	97

Descriptive Statistics

Dependent Variable: CHECK1

pathologic_M	Mean	Std. Deviation	N
M0	8.84887	.851403	80
M1	8.78880	1.064012	5
MX	8.73000	.779362	97
Total	8.78387	.816694	182

Tests of Between-Subjects Effects

Dependent Variable: CHECK1

Source	Type III Sum of Squares	df	Mean Square	F	Sig.
Corrected Model	.620[a]	2	.310	.462	.631
Intercept	3120.401	1	3120.401	4650.514	<.001
pathologic_M	.620	2	.310	.462	.631
Error	120.105	179	.671		
Total	14163.179	182			
Corrected Total	120.725	181			

a. R Squared = .005 (Adjusted R Squared = -.006)

Between-Subjects Factors

		N
pathologic_T		1
	T1	7
	T2	24
	T3	146
	T4	3
	TX	1

Descriptive Statistics

Dependent Variable: DCAF8

pathologic_T	Mean	Std. Deviation	N
	12.14000	.	1
T1	12.01286	.557576	7
T2	11.59417	.422548	24
T3	11.61568	.565024	146
T4	11.47667	.225462	3
TX	11.87000	.	1
Total	11.63011	.545399	182

Tests of Between-Subjects Effects

Dependent Variable: DCAF8

Source	Type III Sum of Squares	df	Mean Square	F	Sig.
Corrected Model	1.475[a]	5	.295	.992	.424
Intercept	1980.355	1	1980.355	6655.999	<.001
pathologic_T	1.475	5	.295	.992	.424
Error	52.365	176	.298		
Total	24671.061	182			
Corrected Total	53.840	181			

a. R Squared = .027 (Adjusted R Squared = .000)

Between-Subjects Factors

		N
pathologic_N		1
	N0	50
	N1	122
	N1b	4
	NX	5

Descriptive Statistics

Dependent Variable: DCAF8

pathologic_N	Mean	Std. Deviation	N
	12.00000	.	1
N0	11.77080	.578301	50
N1	11.57566	.541907	122
N1b	11.51250	.177834	4
NX	11.57200	.257138	5
Total	11.63011	.545399	182

Tests of Between-Subjects Effects

Dependent Variable: DCAF8

Source	Type III Sum of Squares	df	Mean Square	F	Sig.
Corrected Model	1.560[a]	4	.390	1.321	.264
Intercept	2309.690	1	2309.690	7819.766	<.001
pathologic_N	1.560	4	.390	1.321	.264
Error	52.280	177	.295		
Total	24671.061	182			
Corrected Total	53.840	181			

a. R Squared = .029 (Adjusted R Squared = .007)

Between-Subjects Factors

		N
pathologic_M	M0	80
	M1	5
	MX	97

Descriptive Statistics

Dependent Variable: DCAF8

pathologic_M	Mean	Std. Deviation	N
M0	11.53363	.537609	80
M1	11.71800	.569008	5
MX	11.70515	.543739	97
Total	11.63011	.545399	182

Tests of Between-Subjects Effects

Dependent Variable: DCAF8

Source	Type III Sum of Squares	df	Mean Square	F	Sig.
Corrected Model	1.330[a]	2	.665	2.266	.107
Intercept	5484.406	1	5484.406	18695.454	<.001
pathologic_M	1.330	2	.665	2.266	.107
Error	52.511	179	.293		
Total	24671.061	182			

Corrected Total	53.840	181			

a. R Squared = .025 (Adjusted R Squared = .014)

Between-Subjects Factors

		N
pathologic_T		1
	T1	7
	T2	24
	T3	146
	T4	3
	TX	1

Descriptive Statistics

Dependent Variable: PAX8

pathologic_T	Mean	Std. Deviation	N
	7.00000	.	1
T1	8.89429	1.756774	7
T2	7.86262	1.786929	24
T3	8.43773	1.516516	146
T4	7.98367	2.072395	3
TX	8.97400	.	1
Total	8.36702	1.568832	182

Tests of Between-Subjects Effects

Dependent Variable: PAX8

Source	Type III Sum of Squares	df	Mean Square	F	Sig.
Corrected Model	11.460[a]	5	2.292	.929	.463
Intercept	956.923	1	956.923	388.040	<.001
pathologic_T	11.460	5	2.292	.929	.463
Error	434.023	176	2.466		
Total	13186.751	182			
Corrected Total	445.483	181			

a. R Squared = .026 (Adjusted R Squared = -.002)

Between-Subjects Factors

		N
pathologic_N		1
	N0	50
	N1	122
	N1b	4
	NX	5

Descriptive Statistics

Dependent Variable: PAX8

pathologic_N	Mean	Std. Deviation	N
	7.67200	.	1
N0	8.45156	1.555015	50
N1	8.34526	1.611398	122
N1b	8.57650	1.172985	4
NX	8.02380	1.324591	5
Total	8.36702	1.568832	182

Tests of Between-Subjects Effects

Dependent Variable: PAX8

Source	Type III Sum of Squares	df	Mean Square	F	Sig.
Corrected Model	1.663[a]	4	.416	.166	.956
Intercept	1141.034	1	1141.034	455.056	<.001
pathologic_N	1.663	4	.416	.166	.956
Error	443.820	177	2.507		
Total	13186.751	182			
Corrected Total	445.483	181			

a. R Squared = .004 (Adjusted R Squared = -.019)

Between-Subjects Factors

		N
pathologic_M	M0	80
	M1	5
	MX	97

Descriptive Statistics

Dependent Variable: PAX8

pathologic_M	Mean	Std. Deviation	N
M0	8.40966	1.673142	80
M1	7.82140	.627245	5
MX	8.35997	1.517666	97
Total	8.36702	1.568832	182

Tests of Between-Subjects Effects

Dependent Variable: PAX8

Source	Type III Sum of Squares	df	Mean Square	F	Sig.
Corrected Model	1.639[a]	2	.819	.330	.719
Intercept	2714.065	1	2714.065	1094.568	<.001
pathologic_M	1.639	2	.819	.330	.719
Error	443.844	179	2.480		
Total	13186.751	182			
Corrected Total	445.483	181			

a. R Squared = .004 (Adjusted R Squared = -.007)

Between-Subjects Factors

		N
neoplasm_histologic_grade	G1	31
	G2	97
	G3	50
	G4	2
	GX	2

Descriptive Statistics

Dependent Variable: ATF

neoplasm_histologic_grade	Mean	Std. Deviation	N
G1	10.98997	.607894	31
G2	11.12504	.596186	97

G3	11.15164	.652773	50
G4	9.28000	1.400071	2
GX	10.55500	2.283955	2
Total	11.08280	.668959	182

Tests of Between-Subjects Effects

Dependent Variable: ATF

Source	Type III Sum of Squares	df	Mean Square	F	Sig.
Corrected Model	7.735[a]	4	1.934	4.671	.001
Intercept	2653.747	1	2653.747	6411.224	<.001
neoplasm_histologic_grade	7.735	4	1.934	4.671	.001
Error	73.264	177	.414		
Total	22435.787	182			
Corrected Total	80.999	181			

a. R Squared = .095 (Adjusted R Squared = .075)

Between-Subjects Factors

		N
neoplasm_histologic_grade	G1	31
	G2	97
	G3	50
	G4	2
	GX	2

Descriptive Statistics

Dependent Variable: CHECK1

neoplasm_histologic_grade	Mean	Std. Deviation	N
G1	8.35806	.665670	31
G2	8.75959	.763114	97
G3	9.07882	.851412	50
G4	8.72700	2.012426	2
GX	9.24450	1.224002	2
Total	8.78387	.816694	182

Tests of Between-Subjects Effects

Dependent Variable: CHECK1

Source	Type III Sum of Squares	df	Mean Square	F	Sig.
Corrected Model	10.458[a]	4	2.615	4.197	.003
Intercept	1835.940	1	1835.940	2947.051	<.001
neoplasm_histologic_grade	10.458	4	2.615	4.197	.003
Error	110.267	177	.623		
Total	14163.179	182			
Corrected Total	120.725	181			

a. R Squared = .087 (Adjusted R Squared = .066)

Between-Subjects Factors

		N
neoplasm_histologic_grade	G1	31
	G2	97
	G3	50
	G4	2

	GX	2

Descriptive Statistics

Dependent Variable: DCAF8

neoplasm_histologic_grade	Mean	Std. Deviation	N
G1	11.62323	.570014	31
G2	11.62619	.493179	97
G3	11.67320	.581130	50
G4	10.40000	.410122	2
GX	12.08000	.707107	2
Total	11.63011	.545399	182

Tests of Between-Subjects Effects

Dependent Variable: DCAF8

Source	Type III Sum of Squares	df	Mean Square	F	Sig.
Corrected Model	3.527[a]	4	.882	3.102	.017
Intercept	3101.036	1	3101.036	10909.320	<.001
neoplasm_histologic_grade	3.527	4	.882	3.102	.017
Error	50.313	177	.284		
Total	24671.061	182			
Corrected Total	53.840	181			

a. R Squared = .066 (Adjusted R Squared = .044)

Between-Subjects Factors

		N
neoplasm_histologic_grade	G1	31
	G2	97
	G3	50
	G4	2
	GX	2

Descriptive Statistics

Dependent Variable: PAX8

neoplasm_histologic_grade	Mean	Std. Deviation	N
G1	7.75065	1.633894	31
G2	8.45976	1.501131	97
G3	8.68114	1.537438	50
G4	6.87500	.207889	2
GX	7.06150	2.792365	2
Total	8.36702	1.568832	182

Tests of Between-Subjects Effects

Dependent Variable: PAX8

Source	Type III Sum of Squares	df	Mean Square	F	Sig.
Corrected Model	25.406[a]	4	6.352	2.676	.033
Intercept	1418.844	1	1418.844	597.832	<.001
neoplasm_histologic_grade	25.406	4	6.352	2.676	.033
Error	420.077	177	2.373		
Total	13186.751	182			
Corrected Total	445.483	181			

a. R Squared = .057 (Adjusted R Squared = .036)
